# Proceedings of the Twenty-First International Society of Sports Nutrition (ISSN) Conference and Expo

**DOI:** 10.1080/15502783.2024.2374669

**Published:** 2024-07-05

**Authors:** Chad M. Kerksick, Guillermo Escalante, Bill Campbell, Douglas Kalman, Jose Antonio

**Affiliations:** a College of Science, Technology, and Health, Lindenwood University, St, Exercise and Performance Nutrition Laboratory, Kinesiology Department, St Charles, MO, USA; b California State University, San Bernardino, Department of Kinesiology, San Bernardino, CA, USA; c College of Education, Performance & Physique Laboratory, University of South Florida, Tampa, FL, USA; d Nova Southeastern University, Department of Health and Human Performance, Davie, FL, USA

**Keywords:** Exercise, nutrition, dietary supplement, health, performance

**Effect of Kefir Versus Milk on Running Time-Trial Performance in Endurance Master Athletes**

Kristen N. Gross*, Patrick S. Harty, Joesi M. Krieger, Petey W. Mumford, Kyle L. Sunderland, Anthony M. Hagele, and Chad M. Kerksick

Exercise and Performance Nutrition Laboratory, College of Science, Technology, and Health, Lindenwood University, St. Charles, MO USA

Corresponding author: Kristen Gross MS, CISSN: kristen.n.gross@gmail.com

**Background**: Kefir is a fermented milk that contains probiotic strains. Probiotics have been shown to be beneficial to athletes by protecting both the immune and gastrointestinal systems. This study compared flavored kefir (KFR) versus flavored milk (MLK) as a recovery drink in endurance master athletes.

**Methods**: Using a randomized, placebo-controlled, non-blinded crossover design, 11 healthy males (n = 9) and females (n = 2) (55 ± 8 years, 177.3 ± 8.0 cm, 78.2 ± 14.3 kg, 24.7 ± 3.2 kg/m^2^, 43.7 ± 6.9 mL/kg/min) completed three testing visits ingesting either KFR, MLK, or water as placebo (PLA). KFR supplementation occurred for 14 days before the KFR-testing day followed by a 3-week washout period before proceeding to other conditions as randomized. Testing visits consisted of a glycogen depletion (GD) bout, a 4-hour rest period (RP) and a treadmill 5-kilometer time-trial (TT). The Gastrointestinal Symptom Rating Scale (GSRS) survey was assessed at baseline, post-GD, post-RP, and post-TT.

**Results**: No significant difference was found between groups in TT performance in minutes (min) (PLA: 33:39.1 ± 6:29.0, FKF: 33:41.1 ± 5:44.4, and FML: 33:36.2 ± 6:40.5, p = 0.99). GSRS scores were lower, but not significant for KFR compared to both MLK and PLA in four out of the five GSRS dimensions. However, the KFR GSRS total score was lower than PLA after GD (p = 0.005) and non-significantly lower after TT, as well as non-significantly lower than MLK at all timepoints. Blood was collected at baseline and after TT and analyzed for I-FABP (n = 7). KFR levels were non-significantly higher (p > 0.05) than the other conditions.

**Conclusion**: KFR ingestion maintained TT performance and reduced subjective GI symptoms surrounding exercise when compared to MLK or PLA.

**Acknowledgments**: The authors would like to thank the study participants for their time and commitment to this tough protocol that they completed. Also, the authors would like to thank Lifeway Kefir, LLC, for graciously donating the kefir used in this trial. The donor played no role in collecting or analyzing the data. The Exercise and Performance Nutrition Laboratory at Lindenwood University provided funding to cover project expenses, participant compensation, and biomarker reagents.

**Beyond the Buzz: Do Energy Drinks Offer More Than Caffeine for Mental and Physical Tasks?**

Robert Rocanelli^a^, Cassandra Evans^b^, Flavia Pereira^,b,c^, Jose Rojas^b,c^, Jason Curtis^c^, Alyana Andal^a^, Hena Thakker^a^, Cesar Castillo Rodriguez^a^, Juan Carlos Santana^d^, Lia Jiannine^a^, Kristiina Kinnunen^d^, Jose Antonio^a,d^

^a^Department of Health and Human Performance, Nova Southeastern University, Davie, FL, USA; ^b^Department of Health and Human Performance, Rocky Mountain University, Provo, Utah, USA; ^c^Department of Exercise & Sport Science, Keiser University, West Palm Beach, FL, USA; ^d^Institute of Human Performance, Boca Raton, FL, USA.

**Background**: The purpose of this investigation was to assess the effects of an energy drink vs. a positive control drink (i.e. an equal amount of caffeine) on mental and physical tasks.

**Methods**: In a randomized, counterbalanced, crossover trial, 21 exercise-trained volunteers (9 men and 12 women) participated in this study (mean ± SD: age 22 ± 5.9 years; height: 170.8 ± 10.8 cm; weight: 71.9 ± 14.8 kg; body fat percentage: 20.2 ± 9.4; average years of training: 9.5 ± 5.9 years; average daily caffeine intake: 200.5 ± 140.2 mg). Research participants first completed a battery of tests, including the Profile of Mood States (POMS), Psychomotor Vigilance Test (PVT), handgrip strength (HG), and a 1-minute maximum pushup test (PU). Subsequently, they consumed either an energy drink (ED) (Gorilla Mind®) or a positive control drink containing 200 mg of caffeine (CAF) in a randomized, counterbalanced order. After 45 minutes, the entire battery of tests was repeated. Following a one-week washout period, participants returned for a second visit, where they crossed over to consume the other drink and completed the same testing protocol.

**Results**: The delta score for the PVT was −13 ± 19 msec and −5 ± 28 msec. Thus, both groups improved; however, it was not statistically significant (p = 0.3391). Statistical analysis revealed no significant differences between the ED and CAF groups for other outcome measures (POMS: p = 0.152, HG: p = 0.152, PU: p = 0.209).

**Conclusions**: This study found that, in a group of active men and women, consuming an energy drink did not produce significantly different effects on reaction time, mood, upper body muscular endurance, or handgrip strength compared to consuming a drink with an equivalent amount of caffeine alone. These findings suggest that, for these specific measures, the additional ingredients in the energy drink may not have a substantial impact beyond the effects of caffeine. It should be noted that the dose of caffeine was, on average, less than 3.0 mg/kg body mass.

**Disclosures**: This study was unfunded. Jose Antonio is the CEO of the ISSN. The ISSN occasionally receives grant support from companies that manufacture, market and sell caffeine-containing dietary supplements.

**The effect of Tongkat Ali supplementation in healthy exercise-trained males and females**

Jose Rojas^b^, Cassandra Evans^a^, Flavia Pereira^b^, Hena Thakkar^a^, Viraaj Miriyala^a^, Robert Rocanelli^a^, Cesar Castillo^a^, Alyana Andal^a^, Juan Carlos Santana^c^, Lia Jiannine^a^, Kristiina Kinnunen^a^, Jason Curtis^b, d^, and Jose Antonio^a,c^

^a^Exercise and Sport Science, Nova Southeastern University, Davie, FL, USA; ^b^Exercise and Sport Science, Keiser University, West Palm Beach, FL, USA; ^c^Institute of Human Performance, Boca Raton, FL, USA; ^d^Kinesiology Department, Concordia University, St. Paul, MN, USA

Corresponding author: issn.sports.nutrition@gmail.com

**Background**: It has been suggested that supplementation with Tongkat Ali (i.e. the botanical name is Eurycoma longifolia) may affect testosterone concentrations. Whether this also affects body composition is unknown. Thus, the purpose of this investigation was to determine if four weeks of Tongkat Ali (400 mg daily dose) supplementation affected body composition and salivary free testosterone concentrations.

**Methods**: Thirty-three exercise-trained males (n = 19) and females (n = 14) volunteered for this investigation (mean ± standard deviation (SD): age 33.1 ± 13.0 years; height 171.1 ± 11.3 cm; body mass 77.4 ± 16.8 kg; average total years of training 13.9 ± 13.2; average hours of resistance training/week 4.2 ± 2.5; average hours of aerobic exercise/week 3.4 ± 2.8; average hours of other exercise/week 1.1 ± 2.0). Research participants were pre – and post-tested for body composition (InBody 270), Profile of Mood States (POMS), handgrip strength, and sleep (Pittsburgh Sleep Quality Index [PSQI]). In a subset of the research participants, saliva samples were collected and analyzed for cortisol and free testosterone. The research participants consumed 400 mg of Tongkat Ali or a placebo (rice flour) daily for four weeks. An independent t-test was used to analyze the data (i.e. compared the delta scores of the treatment and placebo groups). A p-value of <0.05 was considered significant. The statistical analysis was performed via GraphPad Software (Prism 10).

**Results**: There was no between-group difference in body composition regarding the delta score (mean±SD: lean body mass – Tongkat Ali LBM −0.5 ± 1.9 kg, Placebo −0.4 ± 0.7 kg (p = 0.8218); fat mass – Tongkat Ali −0.5 ± 1.6 kg, Placebo 0.3 ± 1.0 kg (p = 0.0956); percent body fat – Tongkat Ali −0.1 ± 1.4, Placebo 0.5 ± 1.1 (p = 0.2014). Moreover, the other assessments had no between-group differences (e.g. handgrip strength, mood, sleep, and salivary free testosterone and cortisol).

**Conclusions**: Four weeks of supplementation with Tongkat Ali in exercise-trained males and females does not affect body composition, mood, sleep, vigilant attention, handgrip strength, cortisol, and free testosterone.

Acknowledgments: This study was partially funded with an in-kind donation from Nutrition Formulators, Miramar, Florida.

**Low Energy Availability Prevalence, Sleep Quality, and Dietary Habits in Female ROTC Cadets**

Hannah K. Eberhardt^a^, Brandon D. Willingham^b^, Matthew F. Brisebois^a^, Patrick G. Saracino^a^

^a^Department of Human Performance and Health, The University of South Carolina Upstate, Spartanburg, SC, 29303^b^Department of Kinesiology, Coastal Carolina University, Conway, South Carolina, 29526

Corresponding author: Saracinp@uscupstate.edu

**Background**: The primary aim of this study was to determine low energy availability (LEA) prevalence, sleep quality, and dietary habits in female Army Reserve Officer Training Corps (ROTC) cadets.

**Methods**: Following an overnight fast, height, weight, and body composition via Bod Pod were measured. The Low Energy Availability in Females Questionnaire (LEAF-Q) assessed LEA symptoms, while the Pittsburgh Sleep Quality Index (PSQI) and Athlete Sleep Behavior Questionnaire (ASBQ) assessed sleep quality and sleep behavior. To quantify energy expenditure (EE), participants were fitted with a hip-worn physical activity monitor for 7 continuous days. During this time, daily energy intake (EI) was assessed and evaluated relative to the Military Dietary Reference Intakes (MDRIs). As this was a free-living study, participants were asked not to alter their normal behaviors.

**Results**: Nine female ROTC cadets (22 ± 4 yrs, 166.7 ± 6.0 cm, 65.8 ± 8.5 kg, 25.6 ± 6.3% body fat, 48.6 ± 4.6 kg FFM) participated in the study. Mean EA was 32.3 ± 12.2 kcals·kg^−1^ FFM. Three cadets (33.3%) presented with LEA (< 30 kcals·kg^−1^ FFM) and another five cadets (55.6%) did not experience LEA but remained suboptimal (≤ 45 kcals·kg^−1^ FFM). LEAF-Q scores (8.6 ± 4.3) suggest that 66.7% of cadets are at risk for LEA. PSQI scores (6.3 ± 3.3) suggest that 44.4% of cadets experience poor sleep quality. ASBQ scores (38.6 ± 5.9) suggest that 33.3% of cadets experience poor sleep behavior. Cadets consumed 2,052 ± 684 kcals·d^−1^ with an activity EE of 476 ± 116 kcals·d^−1^. Dietary analysis indicated that 33.3%, 44.4%, 88.9%, 22.2% of cadets met the MDRIs for calories (33.3 g·kg^−1^), carbohydrate (4 – 8 g·kg^−1^), protein (0.8 – 1.6 g·kg^−1)^, and fat (20 – 30% total calories), respectively. Those with LEA trended toward greater body fat percentage (p = 0.072) and fat mass (p = 0.094) and consumed less CHO (p < 0.01) and fat (p < 0.05) compared to those without. No differences in sleep parameters were observed.

**Conclusions**: In the present study, all but one cadet was in a low or suboptimal EA state (88.9%). Additionally, 44.4% of female cadets presented with sleep disturbances, and many cadets failed to meet the MDRIs for energy and macronutrient intake.

**Acknowledgments**: This research was funded by the USC Upstate Office of Sponsored Awards and Research Support and by a RISE grant from the Office of the Vice President for Research at the University of South Carolina.

**The effects of turkesterone supplementation on body composition in healthy exercise-trained males and females – a preliminary investigation**

Cesar Castillo^a^, Lia Jiannine^a^, Savannah Calleja^a^, Alyana Andal^a^, Ava Lukowiak^a^, Tobin Silver^a^, and Jose Antonio^a^

^a^Exercise and Sport Science, Nova Southeastern University, Davie, FL, USA

Corresponding author: Jose Antonio issn.sports.nutrition@gmail.com

**Background**: It has been suggested that supplementation with turkesterone, a type of ecdysteroid, may affect testosterone concentrations. Whether this also affects body composition is unknown. Thus, the purpose of this investigation was to determine if four weeks of turkesterone (500 mg daily dose) supplementation affected body composition in healthy males and females.

**Methods**: Thirty-one active males (n = 14) and females (n = 17) volunteered for this investigation. Research participants were assessed pre and four weeks post for body composition (i.e. DXA scan) and handgrip strength. After pre-testing, they were randomized into a placebo (rice flour) or treatment group (i.e. 500 mg per day of Turkesterone [Ajuga Extract]; Nutrition Formulators, Miramar, FL). Subjects were instructed to maintain the same diet and exercise habits during the study.

**Results**: There were no between-group differences (p > 0.05) at baseline for age, height, or body mass. There were no between-group differences in the delta score between the turkesterone and placebo groups in body mass (p = 0.38), lean body mass (p = 0.68), fat mass (p = 0.06), or percent body fat (p = 0.14), nor did it affect handgrip strength (p = 0.9566); (delta score, mean±SD: body mass kg – treatment −0.4 ± 1.8, placebo 0.1 ± 1.8; lean body mass kg – treatment −0.6 ± 1.4, placebo −0.3 ± 1.7; fat mass kg – treatment 0.1 ± 0.6, placebo 0.5 ± 0.6, % fat treatment 0.3 ± 0.6, placebo 0.7 ± 0.9; handgrip – kg treatment 5.0 ± 5.0, placebo 5.2 ± 9.7).

**Conclusions**: Four weeks of supplementation with 500 mg of turkesterone did not affect body composition or strength in active, healthy males and females.

**Acknowledgments**: This study was funded in part by an in-kind donation from Nutrition Formulators, Miramar, Florida.

**Disclosures**: Jose Antonio is the CEO of the ISSN, an academic nonprofit 501c3 organization. The ISSN has received funding from companies that manufacture, market, and sell a variety of dietary supplements.

**Betaine supplementation does not significantly impact exercise performance in the heat**

Jake J. Heydon^a^, Cameron M. McCarthy^b^, Liliana I. Renteria^b^, Kieran G.P. Paterson^b^, Daniel F. Eurich^b^, Timothy D. Griest^b^, Michael J. Ormsbee^b^, Brandon D. Willingham^a,b^

^a^Coastal Carolina University, 100 Chanticleer Dr. E Conway, SC, USA; ^b^Florida State University, 600 West College Ave, Tallahassee, FL, USA

Corresponding Author: bwillingh@coastal.edu

**Background**: Prolonged exercise in heat elevates the risk of heat-related illnesses and diminishes performance. Betaine (BET) has shown promise as an osmoprotectant in animals experiencing heat stress, but human studies are limited. The purpose of this study was to investigate the effects of preloaded BET supplementation on endurance-trained men subjected to active heat stress.

**Methods**: Eight endurance trained men (Age: 26.4 ± 6.8 years; VO_2_peak: 55 ± 4.8 ml·kg^−1^·min^−1^) underwent a double-blind, randomized, crossover study consuming BET (50 mg·kg^−1^, twice per day) or placebo (PLA) for 7 days. The experimental condition consisted of cycling at 70% VO_2_peak for 60 min in the heat (33°C, 35% RH), followed by a supramaximal sprint to exhaustion (120% peak power output). Measures of gas exchange (VO_2_, VCO_2_, and RER), RPE, and blood lactate were assessed across time via repeated measures analysis of variance. Paired-samples T-test were used to determine differences in sprint performance time to exhaustion.

**Results**: No significant differences were found between conditions for measures of gas exchange (VO_2_, VCO_2_, and RER) (p > 0.05). Blood lactate significantly increased across time (p < 0.001), yet no significant differences were detected between conditions at min 30 (PLA: 2.69 ± 1.13 mmol·L^−1^; BET: 2.49 ± 1.05 mmol·L^−1^; p = 0.420) or min 60 (PLA: 5.70 ± 1.83 mmol·L^−1^; BET: 5.23 ± 1.60 mmol·L^−1^; p = 0.353). RPE increased across time (p < 0.001), without significant differences between conditions at any time point (p = 0.938). Sprint performance was not significantly different between conditions (PLA: 115.8 ± 66.0 s; BET: 93.49 ± 35.1 s; p = 0.287).

**Conclusions**: 7 days of BET supplementation did not significantly impact VO_2_, VCO_2_, RER, lactate, or sprint performance when cycling in the heat.

**Acknowledgements**: This study was funded by NOW Foods (Bloomingdale, IL). The funder played no role in data acquisition, data management, nor data interpretation.

**The effects of sleep hygiene education and tart cherry juice supplementation on sleep quality in female collegiate athletes**

Jenna Downey^a^, Jake Heydon^a^, Brandon Willingham^a^

^a^Coastal Carolina University, 100 Chanticleer Dr. E, Conway, SC, USA

Corresponding Author: bwillingh@coastal.edu

**Background**: Sleep hygiene education has become the standard of practice for promoting sleep in collegiate and professional athletes. Likewise, tart cherry juice (TCJ) is known to promote sleep quality in athletic populations. The purpose of this study was to determine if TCJ supplementation provides an additive benefit by enhancing measures of sleep quality compared to sleep hygiene education alone.

**Methods**: 16 female collegiate beach volleyball athletes completed this 21-day study. Week 1 served as baseline with no intervention. At the beginning of Week 2, (day 8), a sleep physician implemented sleep hygiene education. At the beginning of Week 3, (day 15), researchers reinforced sleep hygiene practices and provided TCJ (240 ml, twice daily for seven days). Each week objective- (i.e. wrist-worn actigraphy) and subjective- (i.e. Pittsburgh Sleep Quality Index, PSQI; and Athlete Sleep Behavior Questionnaire, ASBQ) measures of sleep quality were recorded.

**Results**: Regarding objective sleep quality, time in bed (TIB) was significantly increased from Week 1 (445.7 ± 46.2 min) to Week 2 (467.6 ± 33.7 min; p = 0.018), but not from Week 1 to Week 3 (446.3 ± 39.7 min; p = 1.000). Total sleep time (TST) was significantly increased from Week 1 (382.5 ± 46.6 min) to Week 2 (403.4 ± 42.3 min; p = 0.010), but not from Week 1 to Week 3 (379.0 ± 37.7 min; p = 1.000). Regarding subjective sleep quality, PSQI scores were significantly improved from Week 1 (5.4 ± 2.3 AU) to Week 2 (4.3 ± 1.9 AU; p = 0.018) and from Week 1 to Week 3 (3.9 ± 2.2 AU; p = 0.024), but no significant differences were detected from Week 2 to Week 3 (p = 0.566). ASBQ scores were significantly improved from Week 1 (42.7 ± 7.7 AU) to Week 2 (38.4 ± 7.3 AU; p = 0.010) and from Week 1 to Week 3 (36.0 ± 6.0 AU; p < 0.001). Compared to baseline, perceived pre-sleep muscle soreness was improved during TCJ supplementation alone (Week 1 vs Week 3: p = 0.041).

**Conclusion**: Subjective measures of sleep quality (i.e. PSQI and ASBQ scores) were improved by sleep hygiene education alone (Week 2) and in combination with TCJ supplementation (Week 3). However, only sleep hygiene education (Week 2) resulted in greater TIB and TST. Interestingly, only TCJ supplementation significantly improved perceived pre-sleep muscle soreness. Sleep hygiene education is a cost-effective, reliable, method to improve sleep quality in this population.

**Acknowledgements**: This research was funded by Conway Medical Center College of Health and Human Performance.

**Measuring Total Energy Expenditure and Physical Activity Level using Doubly Labeled Water Method in Children with Daily Physical Education**

Nahyun Kim^a^, Mio Bae^a^, Kazuko Ishikawa-Takata^b^, Jonghoon Park^1^

^a^Department of Physical Education, Korea University, Seoul, Republic of Korea; ^b^Faculty of Applied Bioscience, Tokyo University of Agriculture, Tokyo, Japan

*Corresponding Authors: kt207460@nodai.ac.jp (Kazuko Ishikawa-Takata) and jonghoonp@korea.ac.kr (Jonghoon Park)

**Background**: Globally, 80% of children and adolescents have insufficient physical activity. Low total energy expenditure (TEE) and physical activity level (PAL, a multiple of resting metabolic rate (RMR)) are an independent risk factor for obesity and metabolic disease among children. The aim of this study was to measure TEE and PAL in children doing physical education class every day using the doubly labeled water (DLW) method. We also measured the intensity and duration of daily physical activity using a 3-axis accelerometer.

**Methods**: Twelve Korean elementary school children (age = 10.5 ± 0.9) participated in this study (7 boys and 5 girls). All children engaged in physical activity time, including daily physical education and outdoor activities during weekdays. TEE was measured using DLW method for 7 days in free-living conditions. RMR was measured by the Douglas Bag method. PAL was calculated by dividing TEE by RMR. Intensity, including sedentary, light, moderate, and vigorous (MVPA) physical activity, were measured using a 3-axis accelerometer.

**Results**: The children participated in physical education class five times per a week and spent 149.6 ± 10.5 minutes per a day in the physical education class. The physical education time included 31.5 ± 7.6 minutes of MVPA. TEE and PAL were 2271.2 ± 593.4 kcal/day and 1.98 ± 0.28, respectively. TEE/kg was 57.3 ± 7.0 kcal/kg/day. During the entire day, including physical education and NEAT time, time spent by MVPA was 61.7 ± 27.6 minutes. There was a significant negative correlation between body fat % and TEE/kg (r = −0.619, *p* = 0.042).

**Conclusion**: Children doing physical education class five days per week had higher TEE and PAL than children doing 1.5 hours of physical education class per week as assessed by our previous study (Park et al., 2018). Time spent by MVPA was also higher than that of children who do not have regular physical education reported by our previous DLW study (Park et al., 2018). Furthermore, our study found that children with higher TEE had a lower fat mass. This study suggests that physical education class five days per week can increase MVPA and PAL, and may help prevent obesity.

**The Effects of a Sports Nutrition Education Intervention on Sports Nutrition Knowledge, Body Composition, and Resting Metabolic Rate in NCAA Division I Athletes**

Keirstin Roose^a^, Kimberly Michelle Singleton^a^, Jamie McAllister-Deitrick^a^, Ashley Whalen^a^, Brandon Willingham^a^, Chad Kerksick^b^

^a^Department of Kinesiology, Coastal Carolina University; ^b^Exercise and Performance Nutrition Laboratory, Lindenwood University

Corresponding Authors: ksingleto@coastal.edu

**Background**: Nutrition is an essential component to maintaining and enhancing athletic performance. It is imperative athletes have adequate energy intake to support optimal body function, fuel physical work, and facilitate repair and recovery. Typically, athletes have a higher energy requirement compared to the general population which can make it difficult for them to consume enough energy. Athletes have shown to have poor sports nutrition knowledge (SNK), leading to their diets being insufficient in total energy, macronutrients, micronutrients, and fluids. The insufficient diets observed among this population ultimately results in problems maintaining energy balance. Therefore, the overall purpose of this study was to investigate the effects of a 10-week sports nutrition education intervention (SNEI) on SNK, body composition, and resting metabolic rate (RMR) within Division I (DI) female soccer players.

**Method**: National Collegiate Athletic Association (NCAA) DI female soccer players (n = 23) participated in the study during in-season training and matches. SNK, body composition, and RMR were measured pre-and post-intervention. SNK was measured using the Abridged Nutrition for Sports Knowledge Questionnaire (ANSKQ), body composition was measured with air displacement plethysmography (Bod Pod), and RMR was measured using Parvo Medics TrueOne 2400. The intervention consisted of a detailed sports nutrition guide highlighting macronutrients, micronutrients, hydration, and supplementation given at the beginning of the sports season. Additionally, based of pre-testing measurements and physical activity factors, athletes were given estimated daily energy intake requirements for total energy (kcal), protein (g), and carbohydrate (g).

**Results**: Paired samples t-test were conducted to compare differences pre- and post-intervention. Following the intervention and sport season, there was not a significant increase (*p* = 0.612) in SNK (pre, 47.1 ± 10.5; post, 48.2 ± 10.1). No significant differences were found in body mass (pre, 63.4 ± 8.5; post, 63.4 ± 8.3), body fat percentage (pre, 23.4 ± 4.9; post, 23.4 ± 5.5), or RMR (pre, 1,604.4 ± 178.6; post, 1,619.7 ± 186).

**Conclusions**: In this intervention study, there was no significant increase in SNK, suggesting this mode of intervention/delivery is not beneficial in improving SNK. However, finding no significant differences in body mass, body fat percentage, and RMR suggests the intervention positively impacted the dietary behavior of the athletes, as athletes typically have difficulty maintaining energy balance and body composition throughout a sports season. Further research on SNEIs is recommended, specifically focusing on the development and implementation of SNEI’s to positively impact SNK. Additionally, assessing dietary behavior among this population is suggested.

**Impact of a Sports Nutrition Education Intervention on Sports Nutrition Knowledge**

Ashley Whalen^a^, Michelle Singleton^a^, Jamie McAllister-Deitrick^a^, Michael Miller^b^, Chad Kerksick^c^

^a^Department of Kinesiology, Coastal Carolina University; ^b^Department of Human Performance and Health Education, Western Michigan University; ^c^Exercise and Performance Nutrition Laboratory, Lindenwood University

Corresponding Authors: ksingleto@coastal.edu

**Background**: Athletes typically have higher activity levels compared to non-athletic populations; however, previous research has shown athletes characteristically do not meet nutritional recommendations for their level of activity. Researchers have suggested athletes are not meeting these recommendations as they have shown to have poor knowledge regarding sports nutrition. Therefore, we assessed the effects of a sports nutrition education intervention on sports nutrition knowledge (SNK) among collegiate club-sport athletes. The participants’ perceptions of their own SNK, as well as where they receive sources of sports nutrition information, were also evaluated. Lastly, we assessed the impact of how the intervention was delivered, specifically the virtual format and frequency of exposure to the intervention.

**Methods**: Forty-five collegiate club-sport athletes completed a four-week intervention study. Participants were randomized into one of three groups; multiple-intervention group (MIG), single-intervention group (SIG), and control group (CG). Participants in the MIG (n = 14) received the virtual sports nutrition education intervention twice over the course of four weeks, SIG (n = 15) received the intervention at baseline, and CG (n = 16) received no intervention. SNK (Abridged Nutrition for Sports Knowledge Questionnaire), perceptions of SNK, and nutrition information sources were assessed as study outcomes.

**Results**: After the intervention, SNK increased in the SIG participants (p = 0.001) while MIG and CG participants exhibited increased SNK scores, but those differences were not statistically significant (MIG: p = 0.18; CG: p = 0.55). The current study showed collegiate club-sport athletes perceive their nutrition knowledge as adequate at baseline, although they presented with poor baseline SNK scores. Lastly, club-sport athletes are primarily receiving nutrition information from family, athletic trainers/strength coaches, and social media.

**Conclusions**: Collegiate club-sport athletes have poor initial SNK, however the implementation of a single sports nutrition education intervention had the potential to significantly increase their SNK. Conversely, the frequency of exposure to the intervention did increase SNK in this study.

**Student-Athlete Perceptions of Body Composition Measurement Instruments: A Pilot Study**

Shelley L. Holden^a^, Brooke E. Forester^a^

^a^University of South Alabama

Corresponding Authors: bforester@southalabama.edu

**Background**

The purpose of this study was to explore collegiate student-athletes’ perceptions of undergoing three common body composition assessments: bioelectrical impedance (BIA; Tanita), digital anthropometry (DA; Fit3D), and hydrostatic weighing (HW).

**Materials and Methods**

Twenty male (n = 8) and female (n = 12) NCAA Division I collegiate athletes (age: 19.6 ± 1.6 yrs.) underwent three body composition assessments in the following order: BIA, followed by DA, and lastly, HW. Immediately following the three body composition assessments, participants completed questionnaires regarding their demographic and perceptions toward the various body composition assessments. Data was analyzed for themes using a phenomenological qualitative design in Nvivo 14 Qualitative Software.

**Results**

Ten (50%) of the participants selected the BIA as their preferred method while 8 (40%) chose HW. Lastly, only 2 (10%) participants selected DA. Four themes emerged from the qualitative data analysis.

*Theme 1 – Ease of use and timely measurements*

Participants reported they were pleased with both the speed of each test and the ease of use. The majority of participants indicated the BIA for example was ‘quick and easy’, followed by the HW, and lastly the DA.

*Theme 2 – Accuracy is important*

Several of the study participants referenced ‘accuracy’ explicitly. An inherent sense of eagerness was evident as participants mentioned they were anxious to see the results of each instrument.

*Theme 3 – Body composition instruments are fun to use*

Nearly all the participants indicated they enjoyed at least one of the instruments and/or provided a general statement of positivity regarding the study as a whole.

*Theme 4 – Sense of curiosity and novelty*

An interesting theme that emerged was the participants’ level of curiosity. Some were interested in simply obtaining results while others were interested in the methodology associated with the instruments.

**Conclusion**

Participants reported a positive experience undergoing these three body composition assessments. The emergent themes revealed participants are genuinely concerned with their data/scores while placing importance on accuracy and ease of use. Health and fitness professionals should find this data useful as it is the first study to explore athletes’ perceptions of completing BIA, DA, and HW body composition assessments.

**The effects of supplementation with one or two doses of whey protein plus leucine versus placebo on individual and group 1-RM responses following eight weeks of resistance training.**

Robert W. Smith, Jocelyn E. Arnett, Dolores G. Ortega, Trevor D. Roberts, Richard J. Schmidt, Glen O. Johnson, and Terry J. Housh

Nutrition and Health Sciences, University of Nebraska-Lincoln, Lincoln, NE, USA

Corresponding Authors: bsmith80@huskers.unl.edu

**Background**: This study examined the effects of one dose of 40 gm of whey protein with a total of 6.2 gm of leucine taken once daily (1PRO+L), 20 gm of whey protein with a total of 6.2 gm of leucine per dose taken twice daily (total of 12.4 gm of leucine daily [2PRO+L]), and placebo (PLA) on leg extension (LE) 1-RM values following 8 weeks of dynamic constant external resistance (DCER) training.

**Methods**: Thirty-nine men (age = 20.6 ± 1.5 yrs; body mass = 82.8 ± 13.8 kg; height = 181.5 ± 7.3 cm) were randomly assigned to consume either 1PRO+L (n = 13), 2PRO+L (n = 12), or PLA (n = 14) in addition to one set to failure at 80% 1-RM LE DCER training 3d·wk^−1^ for 8 weeks. Leg extension 1-RM was assessed before and after the 8 weeks of supplementation and training. Mean differences for LE 1-RM were analyzed using a 3(Group) × 2(Time) mixed factorial ANOVA and a priori planned pairwise comparisons. Individual responses for LE 1-RM were used to examine the proportion of subjects that exceeded a minimal important difference (MID) value calculated as MID = SD_pooled_ × 0.5, where the SD_pooled_ was the pre-supplementation between-subject standard deviation and 0.5 reflects a moderate effect size. The proportional differences between groups were examined with Chi-squared tests.

**Results**: The 3 × 2 ANOVA indicated no significant interaction or main effect for Group, but a significant (*p* < 0.001) main effect for Time. The planned pairwise comparisons indicated significant (*p* < 0.001) improvements in LE 1-RM for 1PRO+L (127.1 ± 33.2 kg vs.169.2 ± 25.9 kg), 2PRO+L (128.9 ± 22.5 kg vs.157.8 ± 19.6 kg), and PLA (131.4 ± 19.2 kg vs.157.4 ± 28.9 kg) from pre-supplementation to post-supplementation. The percent changes in LE 1-RM for 1PRO+L, 2PRO+L, and PLA were 40.8 ± 38.1%, 26.2 ± 30.1%, and 20.1 ± 15.0%, respectively. A significantly (*p* < 0.05) greater proportion of subjects (13 out of 13; 100.0%) in the 1PRO+L group exceeded the MID for LE 1-RM compared to 2PRO+L (58.3%) and PLA (71.4%) groups, but there was no significant (*p* = 0.484) difference in the proportion of subjects that exceeded the MID between the 2PRO+L and PLA group.

**Conclusions**: The analyses for the individual responses in the present study indicated that supplementation with one dose of 40 gm of whey protein with a total of 6.2 gm of leucine plus resistance training for 8 weeks was more effective for increasing LE 1-RM than either supplementation with two doses of 20 gm of whey protein with 6.2 gm of leucine in each dose (total of 12.4 gm of leucine daily) or placebo.

**Acknowledgements**: This study was sponsored by GNC Holdings, LLC (Pittsburgh, PA). The study sponsor played no role in data acquisition, data management, or data interpretation.

**The effect of quercetin and citrulline on cycling time trial performance**

Jennifer A. Kurtz^a^, Jeff Otis^b^, Kathryn Wilson^b^, Jake Grazer^d^, Rafaela Feresin^c^, J. Andrew Doyle^b^, Ryan Middleton^b^, Emma Devis^f,^ Trisha A. VanDusseldorp^e^, Kimberly Fasczewski^a^

^a^Department of Public Health & Exercise Science, Appalachian State University, Boone, NC, 28607; ^b^Department of Kinesiology & Health, Georgia State University, Atlanta, GA, 30303; ^c^Department of Nutrition, Georgia State University, Atlanta, GA, 30303; ^d^Department of Exercise Science and Sport Management, Kennesaw State University, Kennesaw, GA, 30144 ^e^Bonafide Health, LLC p/b JDS Therapeutics, Harrison, NY, 10528; ^f^Department of Physical Therapy, University of Miami, Coral Gables, FL, 33146

Corresponding Authors: kurtzja@appstate.edu

**Background**: There is growing interest in the use of nutrition and dietary supplements to optimize training and time-trial (TT) performance in cyclists. Separately, quercetin (Q) and citrulline (CIT) have been used as ergogenic aids to improve oxygen (VO_2_) kinetics, perceived effort, and cycling TT performance. However, whether the combination of Q and CIT can provide additive benefits and further enhance cycling performance production is currently unknown.

**Methods**: We examined 28-days of Q+ CIT supplementation on TT performance and several performance measures (i.e. mean power, VO_2_, respiratory exchange ratio (RER), and rate of perceived exertion (RPE)). Forty-eight highly trained cyclists were assigned to one of four supplementation groups: (1) Q + CIT (Q: 500 mg, CIT: 3.0 g), (2) Q (500 mg), (3) CIT (3.0 g), or (4) placebo (3.5 g of a zero-calorie flavored crystal light package). Supplements were consumed two times per day for 28 consecutive days. Participants performed a 20-km cycling time-trial race, pre- and post-supplementation to determine the impact of the combined effects of Q + CIT.

**Results**: There were no potential benefits of Q+ CIT supplementation on TT performance and several performance measures. However, there was an improvement in VO_2_ with Q and CIT when consumed alone.

**Conclusions**: Q+ CIT does not seem beneficial for 20-km TT performance; further exploration with a focus on an increase in cycling duration or Q+ CIT combined with additional polyphenols may amplify any perceived bioactive or metabolic effects on cycling performance. The efficacy of Q+ CIT supplementation to improve cycling performance remains ambiguous.

**Rapid Weight Regain is Not Linked to Success in Professional UFC Fighters During Competition**

Antonella Schwarz^a^, Peter Byers^b^, Lauren Stern^c^, Gabriel J. Sanders^d^, Tobin Silver^b^, Jose Antonio^b^, Corey A. Peacock^b^

^a^Department of Health Sciences, Liberty University, Lynchburg VA USA; ^b^Department of Health and Human Performance, Nova Southeastern University, Davie FL USA; ^c^Health Professions Division, Nova Southeastern University, Davie FL USA; ^d^Exercise Science, University of Cincinnati, Cincinnati OH USA

**CONTACT**: cpeacock@nova.edu

**Background**: Although previous research has explored rapid weight loss (RWL) and rapid weight regain (RWRG) in Mixed Martial Arts (MMA) athletes, there is currently a lack of research analyzing this in professional fighters for the Ultimate Fighting Championship (UFC). There is also a lack of literature analyzing how RWL and RWRG in MMA athletes affect performance outcomes, specifically wins and losses. Therefore, the purpose of the present study was to compare weigh-in versus fight-day weight and whether the percentage change influenced winning or losing records in 24 different UFC athletes. We hypothesize the percentage change between weigh-in and fight-day weight will have no influence on success in professional UFC fighters during competition.

**Methods**: 24 professional MMA fighters (30.9 ± 3.9 yrs.; 176.8 ± 10.4 cm) competing for the UFC during a single event held in 2024 were used for this study. Official weigh-in and fight-day weights were both obtained utilizing the commission-calibrated scale. Paired Samples T Tests were utilized to determine if Official Weigh-In and Fight-Weight were different. Additionally, a regression model was used to determine if % change predicted fight outcome (Wins vs Loses). Statistical significance was set at P ≤ 0.05.

**Results**: There is a significant (P < .001) difference between weigh-in and fight-weight in professional MMA (Table 1). Additionally, there was no significant (P = .694) finding that % change was predictive of fight outcome.

**Table 1.** Weigh-in vs fight weight changes.

	OFFICIAL WEIGH-IN (kg)	FIGHT-WEIGHT (kg)	% CHANGE	P-VALUE
N = 24:	69.6 ± 4.1	75.7 ± 17.3	10.8 ± 4.6	P < .001

M± SD, *Significance set at P ≤ 0.05

**Conclusion**: MMA athletes significantly increase their body weight following official weigh-ins, seeking a competitive advantage. However, it was found that weight gain percentages did not impact competitive outcomes for this particular UFC event. Based on this data, it appears that weight gain percentage does not affect success in professional UFC fighters during competition. This may warrant attention from MMA athletes and weight-cut specialists when deciding the rigor of their future weight-cutting techniques.

**Acknowledgments**

N/A

**Relationships between weight and GPS metrics during kickboxing and sparring in professional MMA athletes**

Peter Byers^a^, Antonella Schwarz^b^, Lauren Stern^c^, Gabriel J. Sanders^d^, Anthony Ricci^a^, Jose Antonio^a^, Corey A. Peacock^a^

^a^Department of Health and Human Performance, Nova Southeastern University, Davie FL USA; ^b^Department of Health Sciences, Liberty University, Lynchburg VA USA; ^c^Health Professions Division, Nova Southeastern University, Davie FL USA; ^d^Exercise Science, University of Cincinnati, Cincinnati OH USA

Corresponding Authors: cpeacock@nova.edu

**Background**: The primary aim of this study was to evaluate the relationship between weight and player workload in professional mixed martial artists (MMA) while wearing global positioning system (GPS) accelerometers during kickboxing and MMA training sessions. Previous research surrounding GPS data in mixed martial arts has focused on intra and inter-unit reliability of GPS accelerometers. This study compared PlayerLoad (PL) & PlayerLoad/min (PL/m) between one training session of kickboxing and one training session of MMA sparring.

**Methods**: Eighteen professional MMA [n = 18 male (30.75 ± 4.12 yrs.; 86.42 ± 12.05 kg)] wore a Sports Vector V7 GPS (Catapult Sports, Boston, MA, USA) accelerometer between the T3 and T4 vertebrae and completed one kickboxing session and one MMA sparring session. During these sessions, PL and PL/m were measured using GPS software. PL is the sum of all accelerations across all axes of tri-axial accelerometry. Multiple correlation analyses examined relationships, and significance was set at p < 0.05.

**Results**: A non-significant weak negative correlation (r = – 0.230, p = 0.410) exists between weight and PlayerLoad kickboxing (PLkick), and between weight and PlayerLoad/min kickboxing (PL/mkick) (r = −0.213, p = 0.447). A non-significant weak negative correlation (r = −0.431, p = 0.335) exists between weight and PlayerLoad sparring (PLspar), and between weight and PlayerLoad/min sparring (PL/mspar) (r = −0.485, p = 0.270). A significant strong positive correlation exists between PLkick and PL/mkick (r = .881, P < .001), HRmaxspar and age (r = .923, P = .003), PL/mspar and PLspar (r = .753, P < .001).

**Conclusion**: There is a weak relationship between weight and PL as well as weight and PL/m in kickboxing and MMA training sessions. This study adds to the limited body of research regarding MMA using GPS data. These results may warrant attention by MMA skill coaches to implement load management in professional MMA. Future research should aim to examine the practical implications of using Catapult Sports accelerometry during a fight camp.

**The Acute Effects of Caffeine and Theacrine on Cognitive Performance Following Fatiguing Exercise in Tactical Personnel**

Gianna F. Mastrofini^a^, Blaine S. Lints^a^, Adam T. Harris^a^, Noah K. Nakagawa^a^, Chiaombim E. Marin-Diala^a^, Mackenzie B. Yoder^a^, Alexa J. Chandler^a^, Sten O. Stray-Gundersen^a^, Davis R. Moore^a^, Shawn M. Arent^a^

^a^Arnold School of Public Health, Dept. of Exercise Science, University of South Carolina, Columbia, SC.

Corresponding Authors: giannafm@e-mail.sc.edu

**Background**: Tactical personnel require sustained attention, impulse control, and working memory to perform their daily tasks. Caffeine is commonly used by tactical personnel to maintain performance during fatiguing situations; however, high levels of caffeine can negatively influence critical aspects of cognition such as impulse control and response consistency. Prior research demonstrates theacrine, a purine alkaloid similar in structure to caffeine but with a longer half-life and fewer side effects, improves performance during real-world cognitive-behavioral tasks. The purpose of this study was to evaluate if combining theacrine with low levels of caffeine could optimize cognitive-behavioral performance following fatiguing exercise better than high levels of caffeine alone.

**Methods**: Twenty (M = 16, F = 4, age = 21.6 ± 3.8y) participants were drawn from tactical populations. We utilized a double-blind, randomized, counterbalanced within-subjects design with the following conditions: 300 mg caffeine (CAF), 150 mg caffeine+150 mg theacrine (CTC), and placebo (PLA).

The initial visit consisted of familiarization with both augmented reality and traditional cognitive-behavioral tasks (object hit-and-avoid and 2-back), and determination of VO_2max_. During experimental conditions, participants completed baseline cognitive-behavioral testing, then consumed either CAF, CTC, or PLA. Cognitive-behavioral testing was repeated at 60-min post-supplementation immediately prior to exercise. The exercise bout consisted of 10, one-minute intervals at 90+% VO_2max_, separated by 120-sec intervals at 40% VO_2max_. Cognitive-behavioral testing was repeated immediately following and 30-min post-exercise.

Change scores were computed for each participant by subtracting baseline performance from each subsequent assessment. RM ANOVAs (α = 0.05) were used to evaluate supplement effects, and significant main and interaction effects of condition and time were decomposed using the Bonferroni correction.

**Results**: We observed a main effect of condition for total accuracy, omission, and commission errors (p < 0.001) during the object hit-and-avoid task. Post-hoc testing revealed both CAF and CTC led to participants demonstrating greater total accuracy (ps≤0.01) and fewer omission (ps<0.01) and commission (ps<0.01) errors as compared to PLA. During the 2-back task, both CAF and CTC resulted in greater accuracy than PLA (ps<0.01). However, only CTC enabled participants to maintain response consistency following fatiguing exercise (p < 0.01).

**Conclusions**: These findings suggest that supplementation with CAF or CTC attenuates fatigue-induced decrements in impulse control, attention, and working memory. Furthermore, CTC reduced response variability following fatiguing exercise, which is a potentially critical finding for tactical personnel who must maintain response consistency under demanding conditions. Thus, combining low levels of caffeine with theacrine may provide a notable advantage over just high levels of caffeine for tactical personnel.

**Acknowledgments**: This project was funded by a grant from AFWERX and Momentous awarded to SMA & RDM. This project was also made possible in part by Grant Number T32-GM081740 from NIH-NIGMS award to GFM. Its contents are solely the responsibility of the authors and do not necessarily represent the official views of the AFWERX, Momentous, nor the NIGMS or NIH.

**Polyphenol-Rich Sorghum Bicolor Extract Enhances Strength Recovery Following Eccentric Exercise**

Clara J. Mitchinson^a^, Brian Benitez^a^, Minyoung Kwak^a^, Pasquale J. Succi^a^, and Haley C. Bergstrom^a^

^a^University of Kentucky, Lexington, KY

Corresponding Authors: clara.mitchinson@uky.edu

**Background**: Due to their antioxidant and anti-inflammatory properties, polyphenols have been shown to enhance exercise performance and recovery. A natural variation of sorghum containing high concentrations of polyphenols, including 3-deoxyanthocyanidins, has been identified. The purpose of this study was to determine if a polyphenol-rich sorghum bicolor extract improves the rate of skeletal muscle strength recovery from eccentric exercise.

**Methods**: Twenty-nine men (mean±SD, age = 23.1 ± 3.8 yrs) completed this randomized, double-blind, placebo (PL) controlled trial to examine the effects of a high- (500 mg) or low- (250 mg) dose polyphenol-rich sorghum bicolor extract (ReDaxin^TM^, RedLeaf® Biologics) on strength recovery from eccentric exercise. Following a familiarization session, subjects were randomly assigned to a high-dose (n = 9), low-dose (n = 10), or PL (n = 10) condition and completed an 18-day supplementation period. The eccentric exercise protocol occurred on day 14 ± 1 of the supplementation period and consisted of 6 sets of 10 maximal, isokinetic (30°s^−1^), unilateral repetitions of eccentric-only muscle actions of the non-dominant forearm flexors. Maximal voluntary isometric contraction (MVIC) torque for forearm flexion (averaged across joint angles of 60, 90, and 120°) was measured immediately prior to (baseline), and 24, 48, and 72 hrs after the eccentric protocol. A 3(group) x 4(time) mixed-model ANOVA and *a priori* planned comparisons were used to examine MVIC at 24, 48, and 72 hrs relative to baseline for each group (p < 0.05).

**Results**: There was no group x time interaction (p = 0.246) or main effect for group (p = 0.267). The planned comparisons indicated that relative to baseline (mean±SD: high-dose: 72.5 ± 15.3 N⋅m, low-dose: 65.0 ± 12.1 N⋅m, PL: 66.9 ± 14.8 N⋅m), MVIC was significantly lower (p = 0.001-0.007) at 24 hrs (high-dose: 58.1 ± 12.6 N⋅m, low-dose: 48.6 ± 11.1 N⋅m, PL: 55.2 ± 16.9 N⋅m) for all groups and remained significantly below baseline (p = 0.001-0.041) for the low-dose and PL at 48 (low-dose: 51.7 ± 13.4 N⋅m, PL: 58.1 ± 18.4 N⋅m) and 72 (low-dose: 56.5 ± 14.2 N⋅m, PL: 57.7 ± 18.8 N⋅m) hrs. For the high-dose, however, MVIC was not significantly different from baseline 48 (63.9 ± 12.1 N⋅m, p = 0.068) and 72 (69.7 ± 14.8 N⋅m, p = 0.366) hrs after the eccentric exercise protocol.

**Conclusions**: Supplementation with a high-dose of the polyphenol-rich sorghum bicolor extract resulted in recovery of muscular strength more rapidly than a low-dose and PL following eccentric exercise. This highlights the potential of 500 mg per day of this polyphenol-rich extract to enhance exercise recovery.

**Acknowledgments**: This research was supported by RedLeaf Biologics, Inc. There is an institutional conflict of interest associated with this research that is being managed by the University of Kentucky (University). One of the equity holders and board members of RedlLeaf Biologics, Inc. (RedLeaf) is a University professor and director of a University institute, and that University employee is also related by marriage to an officer of RedLeaf. Further, the same University employee and the Kentucky Agricultural Experiment Station, managed by the University, have a financial interest in the amount of Rednatural seed annually planted by RedLeaf in the form of license payment. The associated University employee is uninvolved in the activities associated with the research described in this abstract.

**Effects of human milk oligosaccharide ingestion on weight loss and health markers II: Health Biomarkers**

Dante Xing, Joungbo Ko, Choongsung Yoo, Jisun Chun, Drew E. Gonzalez, Broderick Dickerson, Megan Leonard, Victoria Jenkins, Ryan Sowinski, Christopher J. Rasmussen, Richard B. Kreider, FISSN*

Exercise & Sport Nutrition Lab, Texas A&M University, College Station, TX 77843, USA

*Corresponding Authors: rbkreider@tamu.edu

**Background**: Human milk oligosaccharides (HMOs) have prebiotic, immunomodulating, and anti-inflammatory properties. 2’-Fucosyllactose (2’-FL) is one of the most prominent HMOs in human milk. Preclinical studies indicate that 2’-FL has prebiotic, anti-inflammatory, and anti-thrombotic properties and may reduce skeletal muscle atrophy during energy restriction. This study assessed the effects of 2’-FL supplementation during an exercise and hypo-energetic weight loss program on preserving muscle mass, strength, and health markers.

**Materials and Methods**: 41 females and males (38.0 ± 13 yrs., 90.1 ± 15 kg, 31.6 ± 6.6 BMI, 36.9 ± 7 %BF) participated in a randomized, double-blind, placebo-controlled clinical trial. All volunteers participated in a monitored walking and diet intervention for 12 weeks designed to promote a − 500 kcal/d energy deficit. After baseline testing, participants were matched on sex and BMI and randomly assigned to supplement their diet with 3 g/d of banana-flavored maltodextrin powder (PLA) or Momstamin 2’-FL (Advanced Protein Technologies Corp., Gyeonggi-do, Republic of Korea). Fasted blood samples were collected to assess for inflammatory cytokines and platelet aggregation, while feces samples were collected to assess intestinal permeability via zonulin levels. Data were analyzed using a general linear model (GLM) multivariate and univariate analysis with repeated measures, pairwise comparisons, and mean changes from baseline with 95% confidence intervals (CI). Differences with *p* < 0.05 were considered statistically significant, while differences with *p* > 0.05 *to p < *0.10 with medium (0.06) to large (>0.14) partial eta squared (η_p_^2^) effect sizes considered statistical tendencies. Means and 95% CI’s completely above or below baseline were considered clinically significant. Data are presented as mean [UL, LL] CIs with observed p-levels.

**Results**: Overall GLM analysis revealed no overall time (*p* = 0.798, η_p_^2^ = 0.095, moderate effect) or group x time effects (*p* = 0.918, η_p_^2^ = 0.078, moderate effect) for the inflammatory cytokines. Analysis of mean changes from baseline revealed no difference between groups; however, IL-4 tended to be lower for the 2’-FL group than the PLA group at week 6 (2’-FL: 0.884 pg/mL [−0.924, 2.692], *p* = 0.329); PLA: −1.304 pg/mL [−3.156, 0.549], *p* = 0.163; difference −2.187 pg/mL [−4.8, 0.4], p = 0.095). Overall GLM analysis revealed time (*p* < 0.091, η_p_^2^ = 0.118, large effect) but no group x time effects (*p* < 0.133, η_p_^2^ = 0.101, large effect) for platelet aggregation. Pairwise analysis revealed that values in the PLA group were significantly higher than 2’-FL after 6 (2.30 ohms [0.65, 4.0), *p* = 0.008) and tended to be higher after 12 weeks (2.04 ohms [−0.09, 4.2), *p* = 0.06). Additionally, mean changes from baseline revealed a slight increase in platelet aggregation at week 6 for the PLA group that was not seen in the 2’-FL group (2’-FL: −0.356 ohms [−1.696, 0.985], *p* = 0.894; PLA: 1.595 ohms [0.515, 2.674], *p* = 0.005). In terms of intestinal permeability, GLM analysis revealed a significant time (*p* < 0.001, η_p_^2^ = 0.173, large effect) but no interaction effect (*p* = 0.476, η_p_^2^ = 0.038, small effect). Analysis of mean changes from baseline revealed that fecal zonulin in the 2’-FL group significantly decreased after 12 weeks (2’-FL: −64.6 [−102.5, −26.7]), *p* = 0.001) while changes in the PLA group tended to decrease (PLA: −34.2 [−73.1, 4.7], *p* = 0.083) although no differences were seen between groups (−30.4 [−84.7, 23.9], *p* = 0.264).

**Conclusions**: Dietary supplementation with 2’-FL (3 grams/d), exercise, and an energy-reduced diet reduced intestinal permeability and platelet aggregation with no differences identified for the inflammatory cytokines. The 2’-FL group has lower platelet aggregation impedance, suggesting less resistance to blood flow compared to the PLA group. In addition, although not significantly different, fecal zonulin levels (a marker of intestinal permeability associated with leaky gut symptoms) were lower in the 2’-FL group which may warrant additional study. Additional research is warranted to evaluate the potential health effects of 2’-FL supplementation in healthy and older populations.

**Acknowledgments**: This study was funded by a grant from Advanced Protein Technologies Corp. (Gyeonggi-do, Republic of Korea).

**Effects of human milk oligosaccharide ingestion on weight loss and health markers I: Body composition and resting energy expenditure**

Joungbo Ko, Choongsung Yoo, Dante Xing, Jisun Chun, Drew E. Gonzalez, Broderick Dickerson, Megan Leonard, Victoria Jenkins, Ryan Sowinski, Christopher J. Rasmussen, Richard B. Kreider, FISSN*

Exercise & Sport Nutrition Lab, Texas A&M University, College Station, TX 77843, USA

*Corresponding Authors: rbkreider@tamu.edu

**Background**: Human milk oligosaccharides (HMOs) have prebiotic, immunomodulating, and anti-inflammatory properties. 2’-Fucosyllactose (2’-FL) is one of the most prominent HMOs in human milk. Preclinical studies indicate that 2’-FL has prebiotic, anti-inflammatory, and anti-thrombotic properties and may reduce skeletal muscle atrophy during energy restriction. This study assessed the effects of 2’-FL supplementation during an exercise and hypo-energetic weight loss program on preserving muscle mass, strength, and health markers.

**Materials and Methods**: 41 females and males (38.0 ± 13 yrs., 90.1 ± 15 kg, 31.6 ± 6.6 BMI, 36.9 ± 7 %BF) participated in a randomized, double-blind, placebo-controlled clinical trial. All volunteers participated in a monitored walking and diet intervention for 12 weeks designed to promote a − 500 kcal/d energy deficit. After baseline testing, participants were matched on sex and BMI and randomly assigned to supplement their diet with 3 g/d of banana-flavored maltodextrin powder (PLA) or Momstamin 2’-FL (Advanced Protein Technologies Corp., Gyeonggi-do, Republic of Korea). Body composition (percent body fat [%BF], fat mass, fat-free mass [FFM], bone mineral content) and anthropometric (body weight and waist and hip circumferences) measures, as well as resting energy expenditure (REE) parameters, were assessed at 0, 6, and 12 weeks of intervention. Data were analyzed using a general linear model (GLM) multivariate and univariate analysis with repeated measures, pairwise comparisons, and mean changes from baseline with 95% confidence intervals (CI). Differences with *p* < 0.05 were considered statistically significant, while differences with *p* > 0.05 to *p < *0.10 with medium (0.06) to large (>0.14) partial eta squared (η_p_^2^) effect sizes considered statistical tendencies. Means and 95% CI’s completely above or below baseline were considered clinically significant. Data are presented as mean [UL, LL] CIs with observed p-levels.

**Results**: Multivariate analysis revealed an overall time effect (*p* < 0.001, η_p_^2^ = 0.417, large effect) with no significant group x time effect (*p* = 0.760, η_p_^2^ = 0.065, moderate effect) for any of the body composition and anthropometric parameters. Analysis of mean changes from baseline revealed no significant differences between treatments in changes in body weight after 6 and 12 weeks. However, after 12 weeks of intervention, participants in the 2’-FL group experienced clinically significant fat loss (−1,642 g [−3,226, −58], *p* = 0.043), while the PLA group had a more modest and non-significant change in fat mass (−512 g [−2,136, 1,110], *p* = 0.527). Similarly, a trend was noted for changes in percent body fat after 12 weeks (2’-FL: −1.19 % [−2.50, 0.22], *p* = 0.096) but not PLA (0.03 % [−1.45, 1.48], *p* = 0.967). Also, some evidence was noted that the 2’-FL group maintained fat-free mass better than the PLA group (2’-FL: −229 g [−1,250, 791], *p* = 0.652); PLA: −983 g [−2,029, 63], *p* = 0.065). Regarding the REE parameters, multivariate analysis revealed an overall time effect (*p* < 0.001, η_p_^2^ = 0.147, large effect) with no significant differences between treatment groups (*p* = 0.534, η_p_^2^ = 0.032, small effect). Pairwise comparison revealed that the respiratory quotient (−0.37 [−0.06, −0.015], *p* = 0.002) was significantly decreased while fat oxidation tended to be higher (9.6 % [−1.7, 20.9], *p* = 0.095) at 6-weeks in the 2’-FL group Analysis of mean changes from baseline revealed that fat oxidation significantly increased in the 2’-FL group after six weeks of supplementation while changes in the PLA group tended to differ (2’-FL: 15.2 % [4.4, 26.0], *p* = 0.007); PLA: 10.3 [0.8, 21.4], *p* = 0.068) with no significant differences between groups (p = 0.52).

**Conclusions**: Dietary supplementation with 2’-FL (3 grams/d) coupled with exercise and an energy-reduced diet promoted more favorable changes in body composition and REE parameters. Although not statistically significant, the 2’-FL group appeared to maintain fat-free mass better than the PLA group while both groups lost weight. In addition, the 2’-FL group observed a clinically significant amount of fat loss with a trend toward greater changes in percent body fat, while the PLA group did not. These differences increased over time, suggesting a longer intervention may show more significant findings. There was also evidence that 2’-FL promoted increased resting fat and decreased resting carbohydrate oxidation, which supports the contention that 2’-FL supplementation may affect fat loss. However, additional research is warranted to evaluate the potential health effects of 2’-FL supplementation in healthy and older populations.

**Acknowledgments**: This study was funded by a grant from Advanced Protein Technologies Corp. (Gyeonggi-do, Republic of Korea).

**Heart Rate Agreement Between Wrist-worn Photoplethysmography and Chest-worn Electrocardiography Devices During Non-steady-state Exercise**

Sten O. Stray-Gundersen, Jacob J. Becker, Monica M. Ryan, Gianna F. Mastrofini, Blaine S. Lints, Alexa J. Chandler, Robert G. Weaver, and Shawn M. Arent

Arnold School of Public Health, Dept. of Exercise Science, University of South Carolina, Columbia, SC.

**Background**: Wrist-worn photoplethysmography (PPG) devices are commonly used to assess heart rate (HR) during exercise. While some PPG devices appear to accurately reflect HR during steady-state exercise, HR agreement between PPG devices and chest-worn electrocardiography (ECG) devices during non-steady-state exercise has not been investigated. Therefore, we assessed HR agreement between two wrist-worn PPG devices and a research-grade chest-worn ECG device during non-steady-state exercise.

**Methods**: Thirty participants (50% female, age = 21.5 ± 2.7 years, BMI = 24.6 ± 1.5 kg·m^−2^) completed two laboratory visits separated by at least 48 hours. Participants performed a graded VO_2_max treadmill test on Visit 1 and a high intensity functional training (HIFT) session on Visit 2. Participants wore one PPG device (Apple Watch Series 8 [AW] and Polar Grit X [PX]) on each wrist and a chest-worn ECG device (PolarH10 [H10]) throughout both visits. PPG device wrist placement (left vs. right) was randomized and counterbalanced. The HIFT protocol consisted of three rounds of five exercises (kettlebell swings, box jumps, burpees, sit-ups, bike sprints) with a 40:20 second work:rest ratio. Pearson-product moment correlations (r) were calculated to quantify the relationship between each PPG device and the H10 while an equivalence test was performed to evaluate HR agreement between each PPG device and the H10 during the VO_2_max test, HIFT session, and each HIFT exercise with an equivalency region of 2.5% (α = 0.05).

**Results**: AW, but not PX, was statistically equivalent to H10 during the VO_2_max test (AW: r = 0.94, p = 0.02; PX: r = 0.67, p = 1.0) and overall HIFT session (AW: r = 0.94, p = 0.01; PX: r = 0.23, p = 1.0). Similarly, only AW was statistically equivalent to H10 during box jumps (AW: r = 0.63, p = 0.02; PX: r = 0.09, p = 1.0) and bike sprints (AW: r = 0.39, p = 0.009; PX: r = 0.23, p = 1.0). However, neither AW nor PX were statistically equivalent to H10 during kettlebell swings (AW: r = 0.55, p = 0.1 vs. r = 0.13, p = 1.0), burpees (AW: r = 0.61, p = 0.08 vs. r = −0.001, p = 1.0), or sit-ups (AW: r = 0.47, p = 0.08 vs. PX: r = 0.16, p = 1.0).

**Conclusions**: AW PPG HR significantly agreed with H10 ECG HR during a VO_2_max test and HIFT session whereas PX HR did not. While the accuracy of PPG devices varies between brands, AW accurately reflects HR during at least some non-steady-state exercises. However, wrist movement may interfere with PPG accuracy. Brand and exercise modality should therefore be considered when interpreting HR or other metrics utilizing HR for calculations, such as energy expenditure. Future studies should investigate agreement between PPG and ECG devices during a variety of exercises and environmental conditions.

**Acknowledgements**: None

**Disclosures**: This project was made possible in part by Grant Number T32-GM081740 from NIH-NIGMS award to GFM. Its contents are solely the responsibility of the authors and do not necessarily represent the official views of the NIGMS or NIH.

**The Prevalence of Metabolic Syndrome and Cardiometabolic Disease Risk Factors in Men and Women Collegiate Track & Field Throwers**

Taylor Browning,^a^ Samuel Headley,^a^ Elizabeth Mullin,^a^ Andrew R. Jagim,^b,c^ Margaret T. Jones,^c,d^ and Jennifer B. Fields^c,e^

^a^Exercise Science and Athletic Training, Springfield College, Springfield, MA, USA; ^b^Sports Medicine, Mayo Clinic Health Systems, La Crosse, WI, USA; ^c^Patriot Performance Laboratory, Frank Pettrone Center for Sports Performance, George Mason University, Fairfax, VA, USA; ^d^Sport, Recreation, and Tourism Management, George Mason University, Fairfax, VA, USA; ^e^Nutritional Sciences, University of Connecticut, Storrs, CT, USA

**Background**: Due to the prioritization of size and lean body mass accrual for their sport demands, track and field (T&F) throwers are a prominent subgroup of athletes that may be at risk for the development of Metabolic Syndrome (MetSyn) and other cardiometabolic disease risk factors. However, limited research exists examining the prevalence of MetSyn in these high-risk athletes.

**Purpose**: Thus, the purpose of this cross-sectional study was to examine the prevalence of MetSyn in collegiate throwers and provide a descriptive summary of risk factors for MetSyn.

**Methods**: National Collegiate Athletic Association Division I male (n = 17) and female (n = 4) T&F throwers participated in this study. Athletes completed a single day of assessments, which included anthropometrics (height, weight, waist circumference, waist-to-height ratio), body composition (body fat %), blood pressure, venous blood draw (HDL, triglycerides, glucose, IL-6, TNF-a, insulin, Homeostasis Model Assessment of Insulin Resistance (HOMA-IR)), and aerobic capacity (VO_2_ max). Athletes were diagnosed with MetSyn if they met 3 of the 5 following criteria: 1) HDL <40 mg/dL in men; <50 mg/dL in women; 2) Fasting triglycerides >150 mg/dL; 3) Fasting plasma glucose >110 mg/dL; 4) Waist circumference >102 cm in men; >88 cm in women; 5) Systolic blood pressure >130 mmHg and/or diastolic blood pressure >85 mmHg. A Mann Whitney U test was run to identify sex differences in components of MetSyn and cardiometabolic profiles. A Fisher’s exact test was used to determine sex differences in the overall prevalence of MetSyn. Pearson correlation coefficients were used to determine associations between BF% and other measured health markers (p < 0.05).

**Results**: 47% and 0% of men and women athletes, respectively, met at least three criteria for the diagnosis of MetSyn; however, no difference was observed in the prevalence of MetSyn between men and women athletes (*χ^2^*(1, *n* = 21) = 3.04, p = 0.131). Table 1 provides a descriptive summary of risk factors for MetSyn. No sex differences were observed in waist circumference, HDL, triglycerides, glucose, IL-g, TNF-a, insulin, blood pressure, or VO_2_ max (p > 0.05). 47% and 0% of men and women athletes, respectively, met at least three criteria for the diagnosis of MetSyn. Excessive waist circumference (men: n = 11 (64.70%); women: n = 1 (25%)), reduced HDL (men: n = 10 (58.82%); women: n = 2 (50%)), and elevated systolic blood pressure (men: 8 (47.06%); women: n = 1 (25%)) were the most common risk factors for MetSyn. BF% was associated with SBP (r = 0.71), DBP (r = 0.74), insulin (r = 0.58), HORA-IR (r = 0.58), triglycerides (r = 0.51), and VO_2_ max (r = 0.79) (p < 0.05).

**Table 1.** Measured cardiometabolic disease risk factors.

	Men (n = 17)	Women (n = 4)	P-value
Age (yrs)	19.7 ± 1.4	19.8 ± 1.0	0.897
Weight (kg)	120.49 ± 17.12	89.63 ± 9.45	0.004*
Height (cm)	183.93 ± 6.12	171.88 ± 1.65	0.001*
BF%	23.63 ± 8.49	30.26 ± 4.31	0.031*
Waist Circumference (cm)	103.76 ± 10.32	85.13 ± 10.14	0.009
Waist-to-Height Ratio	0.56 ± 0.057	0.50 ± 0.06	0.099
HDL (mg/dL)	41.45 ± 13.58	54.51 ± 15.05	0.122
Triglycerides (mg/dL)	128.92 ± 97.26	71.21 ± 28.65	0.355
Glucose (mg/dL)	78.81 ± 15.66	67.53 ± 24.04	0.554
IL-6 (pg/mL)	2.35 ± 1.91	2.12 ± 2.02	0.202
TNF-a (pg/mL)	1.00 ± 0.00	1.25 ± 0.26	0.871
Insulin (pmol/L)	88.54 ± 57.86	53.92 ± 21.31	0.412
Systolic BP (mm Hg)	133.5 ± 14.9	121.3 ± 17.0	0.275
Diastolic BP (mm Hg)	80.2 ± 9.1	75.8 ± 5.0	0.203
VO_2_ max (mL•min^−1^•kg^−1^)	36.65 ± 6.51	36.68 ± 4.27	1.000

*p < 0.05; BF%: Body fat percent; HDL: high density lipoprotein; IL-6: interleukin-6; TNF-a: tumor necrosis factor; BP: blood pressure

**Conclusions**: This study observed a high incidence of MetSyn and other associated adverse cardiometabolic biomarkers in collegiate T&F throwers, creating concern for these athletes when they retire from the sport and likely become less active. Early screenings, awareness, education, and interventions may have favorable effects on long-term athlete health and safety.

**Fueling Focus: Caffeine, mental health, and athletic performance in college athletes**

Eamonn O’Connell, Stephen Cirella Jr., Elizabeth Germain, Disa Hatfield

Department of Kinesiology, University of Rhode Island, South Kingston, RI, USA

Corresponding Authors: Doch@uri.edu

**Background**: Caffeine has ergogenic effects that appeal to athletes such as decreased rating of perceived exertion and increases in perceived energy. Currently, research is limited regarding athletes’ use of caffeine and their perceptions of caffeine’s efficacy on physical performance and mental health. The purpose of this study was to examine the prevalence, motivation, and perceived benefits of caffeine supplementation reported by Rhode Island collegiate athletes.

**Methods**: Two hundred and two Rhode Island NCAA collegiate athletes (age: 20.02 ± 1.24 years; women = 107, men = 95) completed a cross-sectional online survey to assess caffeine use, motivations for use, and mental health outcomes. Means and standard deviations were calculated for demographic information, caffeine frequency, duration of use, caffeine supplement type, reasons for caffeine use, and mental health outcomes such as anxiety and depression. Bivariate correlations were run on scores of the Eating Attitudes Test (EAT-26), Generalized Anxiety Disorder Scale (GAD-7), Patient Health Questionnaire (PHQ-9), Pittsburgh Sleep Quality Index (PSQI) which assesses disordered eating, generalized anxiety, depression, and sleep quality, respectively, on the scores from the Caffeine Expectancy Questionnaire (CaffEQ) which measures perceived beliefs of the effects of caffeine. Alpha level was set *a priori* to p ≤ 0.05.

**Results**: During the past 6 months 38.6% (*n* = 78) of athletes used caffeine daily, while 37.7% (*n* = 76) used caffeine as least once per week. Athletes reported weekly consumption of coffee/tea (*n* = 119, 58.9%), soft drinks/soda (*n* = 42, 20.8%), energy drinks (*n = *46, 22.9%), and caffeinated medications, candy, or gum (*n* = 19, 9.7%). Mental health measures included the EAT-26 (6.55 ± 8.58; below clinical risk), GAD-7 (13.04 ± 5.31; moderate anxiety), PHQ-9 (12.90 ± 4.71; moderate depression), and PSQI (5.31 ± 2.82; poor sleep quality). Self-reported beliefs of caffeine use were measured by the CaffEQ (71.17 ± 32.34). Scores of the EAT-26, GAD-7, and PSQI were significantly correlated (*r* = 0.251, p < 0.001; *r = *0.220, *p* = 0.003; *r* = 0.181, *p* = 0.014, respectively) with scores from the CaffEQ. PHQ-9 scores were not significantly associated with the CaffEQ (*p* = 0.38).

**Conclusion**: The sample reported frequent engagement of caffeine use from coffee/tea, followed by soft drinks/soda and energy drinks. Self-reported scores of eating behavior, generalized anxiety, and sleep quality are correlated with scores on the CaffEQ. Future efforts will further explore these associations between caffeine use, self-reported reasons for use, and mental health outcomes.

**Acknowledgements**: This research was funded by the Rhode Island Foundation- Clean Competition Fund. The funder played no role in data acquisition, data management, nor data interpretation.

**A Caffeine and Theacrine Combination Improves Ocular Control in Tactical Personnel Under Physically Fatiguing Conditions**

Blaine S. Lints, Adam T. Harrison, Gianna F. Mastrofini, Noah K. Nakagawa, Mackenzie B. Yoder, Chimaobim E. Martin-Diala, Sten O. Stray-Gundersen, Alexa J. Chandler, R. Davis Moore, and Shawn M. Arent

Department of Exercise Science, Arnold School of Public Health, University of South Carolina, Columbia, SC

Corresponding Authors: blints@e-mail.sc.edu

**Background**: Frontal cortical control of saccadic eye movement enables rapid, precise visual assessment and decision-making in tasks requiring visual scanning, threat identification, and situational awareness. The fundamental importance of these qualities in high-stakes military operations highlights the need for interventions that mitigate fatigue-induced cognitive impairments. While caffeine is the most widely consumed psychoactive substance in the world, some of its characteristics (e.g. anxiousness, increased micro-saccades) may be detrimental to warfighter operations. Therefore, we propose the use of theacrine, a purine alkaloid similar in structure to caffeine but with a longer half-life and fewer side effects, in addition to a lower caffeine dose, to enhance cognitive performance and resilience under fatigue.

**Methods**: Tactically-trained participants (N = 20; 22% female, age = 22 ± 4y) completed one familiarization and three experimental visits. The initial visit consisted of familiarization with cognitive tasks (anti-saccade task [APS], object hit-and-avoid [OHA]) and determination of VO_2max_. We utilized a double-blind, counterbalanced, placebo-controlled, randomized within-subjects design, with sessions separated by ≥96 hours. Participants completed baseline APS testing, then consumed either 300 mg caffeine [CAF], 150 mg caffeine + 150 mg theacrine [CTC], or placebo [PLA]. The APS was repeated at 60-min post-supplementation immediately prior to exercise. Exercise consisted of 10, one-minute intervals at 90+% VO_2_max, separated by 120-sec intervals at 40% VO_2_max. APS testing was repeated immediately and 30-min post-exercise. Change scores were computed for each participant by subtracting baseline performance from each subsequent assessment. RM ANOVAs (α = 0.05) were used to evaluate ocular performance, and significant interactions were decomposed using the Bonferroni correction.

**Results**: Compared to the PLA condition, CAF exhibited a higher percentage of foveated objects (p = 0.01) during the OHA task.

Coefficient of variation for reaction time (CVRT) to anti-saccade targets was lower in the CTC condition compared to CAF (p < 0.01) and PLA (p < 0.01) 30-min post-exercise.

Compared to the PLA condition, CTC and CAF demonstrated greater total accuracy (CTC & CAF: ps<0.01), lower CVRT to pro-targets (CTC & CAF: ps≤0.03), and lower % anti-target errors (CTC & CAF: ps<0.01), but only the CTC condition demonstrated lower pro-target and anti-target RTs (p ≤ 0.03).

**Conclusions**: Our findings indicate both the CAF and CTC conditions were associated with improvements in ocular performance during continuous and discrete cognitive tasks. Despite the beneficial effects of CAF in object foveation during the OHA task, only CTC outperformed PLA in all APS measures, and those improvements persisted following high-intensity exercise.

**Acknowledgements**: This abstract was made possible by grant funding provided by AFWERX and Momentous, and in part by Grant Number T32-GM081740 from NIH-NIGMS. Its contents are solely the responsibility of the authors and do not necessarily represent the official views of AFWERX, Momentous, the NIGMS or NIH.

**Effects of human milk oligosaccharide ingestion on weight loss and health markers III: Quality of Life and Markers of Safety**

Drew E. Gonzalez, Joungbo Ko, Choongsung Yoo, Dante Xing, Jisun Chun, Broderick Dickerson, Megan Leonard, Victoria Jenkins, Ryan Sowinski, Christopher J. Rasmussen, Richard B. Kreider, FISSN*

Exercise & Sport Nutrition Lab, Texas A&M University, College Station, TX 77843, USA

*Corresponding Authors: rbkreider@tamu.edu

**Background**: Human milk oligosaccharides (HMOs) have prebiotic, immunomodulating, and anti-inflammatory properties. 2’-Fucosyllactose (2’-FL) is one of the most prominent HMOs in human milk. Preclinical studies indicate that 2’-FL has prebiotic, anti-inflammatory, and anti-thrombotic properties and may reduce skeletal muscle atrophy during energy restriction. This study assessed the effects of 2’-FL supplementation during an exercise and hypo-energetic weight loss program on preserving muscle mass, strength, and health markers.

**Materials and Methods**: 41 females and males (38.0 ± 13 yrs., 90.1 ± 15 kg, 31.6 ± 6.6 BMI, 36.9 ± 7 %BF) participated in a randomized, double-blind, placebo-controlled clinical trial. All volunteers participated in a monitored walking and diet intervention for 12 weeks designed to promote a − 500 kcal/d energy deficit. After baseline testing, participants were matched on sex and BMI and randomly assigned to supplement their diet with 3 g/d of banana-flavored maltodextrin powder (PLA) or Momstamin 2’-FL (Advanced Protein Technologies Corp., Gyeonggi-do, Republic of Korea). Fasted blood samples and resting hemodynamics (resting heart rate [RHR] and blood pressure [BP]) were collected to assess the safety of supplementation. Quality of life (QOL) questionnaire and self-reported side effect responses were assessed at 0, 6, and 12 weeks. Continuous data were analyzed using a general linear model (GLM) multivariate and univariate analysis with repeated measures, pairwise comparisons, and mean changes from baseline with 95% confidence intervals (CI). Categorical data were analyzed by Chi-squared analysis. Differences with *p* < 0.05 were considered statistically significant, while differences with *p* > 0.05 *to p < *0.10 with medium (0.06) to large (>0.14) partial eta squared (η_p_^2^) effect sizes considered statistical tendencies.

**Results**: Overall GLM analysis revealed no significant time (*p* < 0.650, η_p_^2^ = 0.158) or group x time effects (*p* < 0.657, η_p_^2^ = 0.158) for white blood cells, red blood cells, hemoglobin, hematocrit, mean corpuscular volume (MCV), mean corpuscular hemoglobin (MCH), mean corpuscular hemoglobin concentration (MCHC), red cell dimension width (RDW), neutrophils, lymphocytes, monocytes, eosinophils, basophils, and platelets. Univariate analysis revealed that hematocrit (*p* = 0.079, η_p_^2^ = 0.064, moderate effect) and MCV values (*p* = 0.056, η_p_^2^ = 0.074) tended to change over time with no group differences noted in the remaining variables. GLM analysis revealed a significant time (*p* < 0.001, η_p_^2^ = 0.179) with no significant group x time effect (*p* = 0.774, η_p_^2^ = 0.021). Pairwise comparison analysis revealed that RHR decreased from baseline after 6 weeks in the 2’-FL (*p* = 0.002), while it took 12 weeks to observe a reduction in RHR in the PLA group (*p* = 0.002). No significant interaction effects were observed in resting BP. The chi-square analysis revealed significant differences between groups in the QOL questions. More favorable responses were observed in the 2’-FL group in ratings *of physical health impact including does health limit vigorous activities (p *< 0.001),* moderate activities (p *= 0.065), ability to carry groceries (*p* = 0.065), ability to climb stairs (*p* < 0.001), ability to bend, kneel or stoop (*p* = 0.003), walk more than a mile (*p* = 0.025), or ability to accomplishing less than they would like (*p* = 0.065). Differences were also noted in emotional-related ability to cut down on the amount of time on work or other activities (*p* = 0.031), ratings of bodily pain (*p* = 0.083), perceptions of having lots of energy (*p* = 0.034), feeling tired (*p* = 0.048), and expectations about health getting worse (*p* = 0.033). No significant differences between treatment groups were observed in the frequency or severity of self-reported side effects.

**Conclusions**: Dietary supplementation with 2’-FL (3 grams/d), exercise, and an energy-reduced diet improved self-reported perceptions of QOL questionnaire responses, particularly the inability to perform physical activity and limitations to physical health. In addition, there is evidence that participants in the 2’-FL group experienced a greater training adaptation regarding resting heart rate compared to the PLA group. Lastly, the 12-week 2’-FL supplementation protocol was well tolerated and not associated with untoward side effects. Additional research is warranted to evaluate the potential health effects of 2’-FL supplementation in healthy and older populations.

**Acknowledgments**: This study was funded by a grant from Advanced Protein Technologies Corp. (Gyeonggi-do, Republic of Korea).

**Pain Perception Responses to Maximal Aerobic Exercise in Males and Females: A Detailed Examination**

Brandi Antonio^a^, Jeffrey R. Stout^a^, Danielle A. Sterner^a^, Abigail Anderson^b^, and David H. Fukuda^a^

^a^Physiology of Work and Exercise Response (POWER) Lab, Institute of Exercise Physiology and Rehabilitation Sciences, University of Central Florida, Orlando, FL, USA; ^b^Rehabilitation and Modulation of Pain (RAMP) Lab, Institute of Exercise Physiology and Rehabilitation Sciences, University of Central Florida, Orlando, FL, USA

Corresponding Authors: Brandi.Antonio@ucf.edu

**Background**:

The primary goal of this study was to explore the effects of a graded exercise test (GXT) on the change in specific pain assessment measures: pressure pain threshold (PPT), pressure pain tolerance (PPTol), heat pain threshold (HPT), temporal summation (TS), and conditioned pain modulation (CPM). Most current research on exercise-induced hypoalgesia (EIH) involves exercise protocols that exceed 10 minutes in duration and 75% of one’s VO2 max but are still considered submaximal. To our knowledge, the effects of a GXT on this comprehensive set of pain measures have not been previously examined.

**Methods**: 31 male and female participants (males: n = 16, height = 177 ± 6 cm, weight = 75.6 ± 6.9 kg,VO2peak = 37.8 ± 7.1 ml/kg/min, females: n = 15, height = 167 ± 7 cm, weight = 66.1 ± 12.4 kg, VO2peak = 29.2 ± 7.3 ml/kg/min) underwent pain sensitivity evaluation (PPT, PPTol, HPT, TS, CPM) both before and after performing a graded exercise test (GXT) to exhaustion on a cycle ergometer. PPT, PPTol, and HPT were applied twice to the dominant forearm and thigh before and after the GXT. TS was applied to the non-dominant palm once, and CPM was applied on the non-dominant foot (pressure stimulus) and the dominant hand (cold water stimulus). Two-way repeated measures ANOVA was used to examine changes in HPT, PPT, and PPTol values before and after the GXT for two different body sites (forearm and thigh) separately for males and females to analyze any potential sex differences in pain perception. The dependent t-test was used to determine if there were changes in TS and CPM in males and females, as both tests are not direct measurements, but rather changes in pre-post measures.

**Results**: Statistical analyses revealed that for PPT, no significant interaction was found between location and time for either sex. However, a significant main effect for location was observed for both males (F(1, 15) = 7.125, p = .018, Partial η^2^ = .322) and females (F(1, 14) = 19.390, p < .001, Partial η^2^ = .581), indicating different pain sensitivity between forearm and thigh. For PPTol, while males did not show an interaction effect, females exhibited a significant interaction (F(1, 14) = 12.871, p = .003, Partial η^2^ = .479), with a significant reduction in PPTol of the forearm (mean difference = 147.9, p = .004) post-GXT. The HPT analysis revealed that there was no interaction for males but a significant interaction for females (F(1, 14) = 5.996, p = .028, Partial η^2^ = .300), with a significant decrease in HPT of the forearm (mean difference = 0.610, p = .022). The TS and CPM measures did not show significant changes after GXT in either sex.

**Conclusions**: These results suggest that acute physical exertion can alter pain perception, with noticeable differences between sexes and anatomical locations. These findings highlight the complexity of pain mechanisms influenced by exercise and underscore the need for further exploration of the physiological and psychological factors involved.

**Effects of supplementation with microalgae containing fucoxanthin on cognitive function in healthy and active late middle-aged individuals experiencing initial signs of memory and cognitive decline II: health and safety markers**

Megan Leonard^a^, Choongsung Yoo^a^, Joungbo Ko^a^, Dante Xing^a^, Jisun Chun^a^, Drew E. Gonzalez^a^, Victoria Jenkins^a^, Broderick Dickerson^a^, Landry Estes^a^, Sarah Johnson^a^, Jacob Broeckel^a^, Jonathan Maury^b^, Rémi Pradelles^b^, Ryan Sowinski^a^, Christopher J. Rasmussen^a^, Richard B. Kreider, FISSN^a^

^a^Exercise & Sport Nutrition Lab, Texas A&M University, College Station, TX 77843, USA; ^b^Research & Development Department, Microphyt, 34670 Baillargues, FRA

Corresponding Authors: rbkreider@tamu.edu

**Background**: An emerging area of active and sports nutrition is helping people maintain physical performance and cognitive function as they age. We reported that supplementation with the microalgae extracts *Phaeodactylum tricornutum* (PT) containing the carotenoid fucoxanthin (4.4 and 8.8 mg/d) enhanced measures of cognitive function in young adult e-gamers. This study examined whether supplementation with PT containing 8.8 mg/d of fucoxanthin affects cognitive function in healthy, late middle-aged, physically active individuals experiencing self-perceived memory and cognitive decline.

**Methods**: Forty-three healthy late middle-aged females and males (64.3 ± 6 years, 79.8 ± 16 kg, 27.0 ± 4 BMI) with non-clinical self-perceptions of memory and cognitive decline and a Mini-Mental State Examination (MMSE) score >24 (28.8 ± 0.9) participated in a randomized, double-blind, placebo-controlled clinical trial. Participants were familiarized with and practiced all cognitive tests to establish reliability. After baseline testing, participants were matched on sex and BMI and randomly assigned to supplement their diet with a placebo (PL) or 1100 mg/day of PT standardized for 0.8% (8.8 mg) fucoxanthin (FX, Brainphyt™, Microphyt, Baillargues, FRA) for 12 weeks while maintaining their regular diet and physical activity levels. Fasting blood samples, resting hemodynamics, and body weight were taken at 0, 4, and 12 weeks of intervention. Data were analyzed using a general linear model (GLM) multivariate and univariate analysis with repeated measures with pairwise comparisons and mean changes from baseline with 95% confidence intervals (CI). Differences with *p* < 0.05 were considered statistically significant, while differences with *p* > 0.05 *to p < *0.10 with medium (0.06) to large (>0.14) partial eta squared (η_p_^2^) effect sizes considered statistical tendencies. Means and 95% CI’s completely above or below baseline were considered clinically significant. Data are presented as mean [UL, LL] CIs with observed p-levels.

**Results**: No differences were found in body weight, renal and liver function markers, and self-reported side effects. GLM analysis revealed an overall time effect (*p* = 0.005, η_p_^2^ = 0.109, medium effect) with no treatment x time effect (*p* = 0.306, η_p_^2^ = 0.043, small effect) for the resting hemodynamic measures. Analysis of mean changes from baseline revealed that resting heart rate decreased during weeks 4 and 12 for the PL group (Week 4: PL −3.95 bpm [−6.65, −1.26], *p* = 0.005, Week 12: −3.38 bpm [−7.05, 0.29], *p* = 0.070) while not significantly changed in the FX group (Week 4: −0.5 bpm [−3.2, 2.1], *p* = 0.704, Week 12: 1.14 bpm [−2.5, 4.7], *p* = 0.526). The difference at Week 4 tended to differ between groups (p = 0.072). Further, the PL group demonstrated an increase in systolic (Week 12: PL 5.9 mmHg [1.7, 9.9], *p* = 0.007; FX −3.6 mmHg [−10.2, 2.9], *p* = 0.269, difference 4.4 mmHg [−1.1, 10.1], *p* = 0.135) and diastolic blood pressure (Week 12: PL 3.05 mmHg [0.8, 5.3], *p* = 0.009; FX −0.4 mmHg [−2.6, 1.8], *p* = 0.710, difference 2.64 mmHg [−0.5, 5.8], *p* = 0.099) while unchanged in the FX group. GLM analysis revealed no overall time (*p* = 0.569, η_p_^2^ = 0.159, large effect) or treatment x time effects (*p* = 0.224, η_p_^2^ = 0.199, large effect) for the whole blood cell counts. Analysis of differences in mean changes from baseline revealed that the PL group had lower white blood cells (Week 4: −0.62 K/μL [−1.2, 0.002], *p* = 0.051) and neutrophils (Week 4: −5.0 % [−9.9, −0.03], *p* = 0.049), higher lymphocytes (Week 4: 3.24 % [−0.32, 6.8], *p* = 0.074) and platelets (Week 12: 18.4 K/μL [1.5, 35.2], *p* = 0.033), than the FX group. GLM analysis revealed no overall time (*p* = 0.181, η_p_^2^ = 0.083, medium effect) or treatment x time effects (*p* = 0.710, η_p_^2^ = 0.044, small effect) for the insulin sensitivity markers. Analysis of mean changes from baseline revealed that insulin (Week 4: −3.91 μU/mL [−7.385, −0.436], *p* = 0.028; Week 12: −4.63 μU/mL [−8.49, −0.77], *p* = 0.020) and HOMA (Week 4: −0.77 [−1.6, 0.08], *p* = 0.076; Week 12: −1.08 [−2.07, −0.09], *p* = 0.033) were lower, while the glucose/insulin ratio (Week 4: 3.14 [−0.13, 6.4], *p* = 0.060; Week 12: 7.29 [−0.72, 15.3], *p* = 0.073) and QUICKI (Week 4: 0.016 [0.003, 0.029], *p* = 0.019; Week 12: 0.020 [0.002, 0.038], *p* = 0.030) were higher from baseline in the FX group while not significantly different from baseline in the PL group. GLM analysis revealed an overall time effect (*p* = 0.050, η_p_^2^ = 0.139, medium effect) with no treatment x time effect (*p* = 0.431, η_p_^2^ = 0.086, medium effect) for the blood lipids. Analysis of mean changes from baseline revealed HDL increased (Week 12: 3.476 mg/dL [0.830, 6.123], *p* = 0.011) while the LDL: HDL ratio decreased (Week −0.181 [−0.350, −0.013], *p* = 0.016) for the PL group while not significantly affected with FX. Lastly, GLM analysis revealed no time effect (*p* = 0.580, η_p_^2^ = 0.110, medium effect) with no treatment x time effect (*p* = 0.648, η_p_^2^ = 0.104, medium effect) for the inflammatory cytokines. Analysis of mean changes from baseline revealed evidence that Il-1β and TNFα increased in the FX group over time while IL-6, IL-10, and IFNγ decreased in the PL group. However, only week 12 TNFα values tended to be higher in the FX group (0.97 pg/mL [−0.09, 2.0], *p* = 0.072).

**Conclusions**: These findings provide evidence that 1100 mg of PT, containing 8.8 mg of FX, improved insulin sensitivity, did not adversely impact resting hemodynamics, did not favorably impact blood lipids or inflammatory markers, and was well tolerated in late middle-aged individuals maintaining regular diet and physical activity patterns who perceive some memory and cognition.

**Acknowledgments**: This study was funded by a grant from Microphyt, Baillargues, France.

**Effects of supplementation with microalgae containing fucoxanthin on cognitive function in healthy and active late middle-aged individuals experiencing initial signs of memory and cognitive decline I: cognitive function**

Choongsung Yoo^a^, Joungbo Ko^a^, Dante Xing^a^, Jisun Chun^a^, Drew E. Gonzalez^a^, Victoria Jenkins^a^, Broderick Dickerson^a^, Megan Leonard^a^, Landry Estes^a^, Sarah Johnson^a^, Jacob Broeckel^a^, Jonathan Maury^b^, Rémi Pradelles^b^, Ryan Sowinski^a^, Christopher J. Rasmussen^a^, Richard B. Kreider, FISSN^a^

^a^Exercise & Sport Nutrition Lab, Texas A&M University, College Station, TX 77843, USA; ^b^Research & Development Department, Microphyt, 34670 Baillargues, FRA

*Corresponding Author: rbkreider@tamu.edu

**Background**: An emerging area of active and sports nutrition is helping people maintain physical performance and cognitive function as they age. We reported that supplementation with the microalgae extracts *Phaeodactylum tricornutum* (PT) containing the carotenoid fucoxanthin (4.4 and 8.8 mg/d) enhanced measures of cognitive function in young adult e-gamers.This study examined whether supplementation with PT containing 8.8 mg/d of fucoxanthin affects cognitive function in healthy, late middle aged, and physically active individuals experiencing self-perceived memory and cognitive decline.

**Methods**: Forty-three healthy late middle-aged females and males (64.3 ± 6 years, 79.8 ± 16 kg, 27.0 ± 4 BMI) with non-clinical self-perceptions of memory and cognitive decline and a Mini-Mental State Examination (MMSE) score >24 (28.8 ± 0.9) participated in a randomized, double-blind, placebo controlled clinical trial. Participants were familiarized with and practiced all cognitive tests to establish reliability. After baseline testing, participants were matched on sex and BMI and randomly assigned to supplement their diet with a placebo (PL) or 1100 mg/day of PT standardized for 0.8% (8.8 mg) fucoxanthin (FX, Brainphyt™, Microphyt, Baillargues, FRA) for 12 weeks while maintaining their regular diet and physical activity levels. Fasting blood samples and cognitive tests were performed at 0, 4, and 12 weeks of intervention. Data were analyzed using a general linear model (GLM) multivariate and univariate analysis with repeated measures, pairwise comparisons, and mean changes from baseline with 95% confidence intervals (CI). Differences with *p* < 0.05 were considered statistically significant, while differences with *p* > 0.05 *to p < *0.10 with medium (0.06) to large (>0.14) partial eta squared (η_p_^2^) effect sizes considered statistical tendencies. Means and 95% CI’s completely above or below baseline were considered clinically significant. Data are presented as mean [UL, LL] CIs with observed p-levels.

**Results**: GLM analysis revealed no overall interaction effects in word recall (*p* = 0.892, η_p_^2^ = 0.022, small effect), word recognition (*p* = 0.325, η_p_^2^ = 0.080, medium effect), choice reaction (*p* = 0.374, η_p_^2^ = 0.039, small effect), picture recognition (*p* = 0.569, η_p_^2^ = 0.064, medium effect), Corsi Block (*p* = 0.607, η_p_^2^ = 0.012, small effect), Stroop test (*p* = 0.610, η_p_^2^ = 0.085, medium effect) or light reaction test (*p* = 0.937, η_p_^2^ = 0.014, small effect) while digit vigilance tended to interact (*p* = 0.052, η_p_^2^ = 0.074, medium effect). Univariate analysis revealed significant time effects in a number of variables and that digit vigilance targets correct (*p* = 0.070, η_p_^2^ = 0.068, medium effect), Stroop words correct (*p* = 0.089, η_p_^2^ = 0.060, medium effect) and incongruent words correct (*p* = 0.087, η_p_^2^ = 0.060, medium effect) tended to interact. Analysis of mean changes from baseline revealed that FX increased recall attempts (Week 4: 0.864 [−0.074, 1.802], *p* = 0.070), correct attempts (Week 4: 1.045 [0.217, 1.873], *p* = 0.015; Week 12: 0.955 [−0.154, 2.063], *p* = 0.090), delayed attempts (Week 4: 0.864 [0.008, 1.719], p = 0.048; Week 12: 1.091 [0.078, 2.104], p = 0.036), and delayed-correct recall (Week 4: 0.818 [−0.019, 1.656], *p* = 0.055) from baseline, while the PL group experienced no effect, indicating an improved working memory and long-term memory transfer. The choice reaction time variables target correct (Week 12: 0.727 [0.002, 1.453], *p* = 0.049), correct response time (Week 12: 49.885 [−9.469, 109.239], *p* = 0.097), and overall response time (Week 12: 52.168 [−7.166, 111.502], *p* = 0.083) increased from baseline with FX while not affected in the PL group. Picture recognition test analysis revealed that FX maintained correct and overall reaction times while the PL group was unaffected. There was also evidence that FX improved the digit vigilance test variables number of targets correctly identified (Week 12: 3.535 [−2.036, 4.865], *p* = 0.072) and correct reaction time (Week 4: 9.391 [0.208, 18.574], *p* = 0.045), while the PL group had increased false alarms (Week 4: 1.238 [−0.077, 2.553], *p* = 0.064). FX also increased the Stroop color-word test variables words correct (Week 4: 4.469 [- 0.863, 9.801], *p* = 0.098) and incongruent words correct (Week 4: 9.091 [−1.563, 19.746], *p* = 0.0092) from baseline. No significant differences were observed between groups or over time in word recognition or the Corsi-block assessment. Lastly, there were similar scores for the light chain reaction test, with no statistically significant differences noted between the groups.

**Conclusions**: These findings provide evidence that 1100 mg of PT, containing 8.8 mg of FX, improved working and secondary memory, attention and vigilance, accuracy, and executive function from baseline in late middle-aged individuals maintaining regular diet and physical activity patterns who perceive some memory and cognitive decline.

**Acknowledgments**: This study was funded by a grant from Microphyt, Baillargues, France.

**The Effect of Acute Essential Amino Acid Intake on Phase Angle and Fluid Shifts Between Healthy and Sarcopenic Adults**

Callie L. Unrein^1^, David D. Church^2^, Arny A. Ferrando^2^, Robert R. Wolfe^2^, Katie R. Hirsch^1^

^1^Department of Exercise Science, Arnold School of Public Health, University of South Carolina. Columbia, SC, 29208, USA; ^2^Center for Translational Research in Aging & Longevity, University of Arkansas for Medical Sciences, Little Rock, AR, 72205, USA

Corresponding Authors: Katie Hirsch (khirsch@mailbox.sc.edu)

**Background**: Raw bioimpedance values, such as phase angle (PhA), are indicators of muscle quality in healthy and clinical populations. Bioelectrical impedance analysis (BIA) is also sensitive to fluid shifts between extracellular (ECW) and intracellular (ICW) compartments, which occur with nutrient uptake into skeletal muscle. These outcomes could identify anabolic resistance, characterized by altered amino acid uptake by skeletal muscle, which contributes to diseases like sarcopenia. The purpose of this study was to evaluate effects of acute essential amino acid (EAA) intake on segmental (R/LArm, trunk, R/LLeg) PhA and fluid distribution (ECW, ICW) in healthy young adults (HYA), healthy older adults (HOA), and sarcopenic older adults (SOA).

**Methods**: As part of a larger metabolic study, 12 participants (4 M; 8 F) were characterized as HYA (n = 5; Age:27.4 ± 1.3 yrs; %BF:33.8 ± 7.5%), HOA (n = 4; Age:74.5 ± 1.5 yrs; %BF:37.9 ± 3.6%), and SOA (n = 3; Age:76.0 ± 1.0 yrs; %BF:34.6 ± 3.2%), defined by age and grip strength (SOA: M < 35.5 kg; F < 20 kg). Using multi-frequency BIA, segmental PhA (degrees at 50 kHz), ICW (L), and ECW (L) were measured every 30 min before (0, 30, 60 min) and after (90, 120, 150, 180, 210, 240, 270, 300 min) EAA consumption (10 g EAA; 10oz water). Repeated measures ANOVAs evaluated group×time interactions, followed by Tukey’s post-hoc analysis.

**Results**: There were no significant interaction or time effects for any outcome (all p = 1.000). There were significant group effects for all PhA segments (p < 0.001). LLeg PhA was greater in HYA than HOA (MD±SE: 1.45 ± 0.48°; p = 0.036) and non-significantly greater than SOA (1.38 ± 0.52°; p = 0.064). Similar non-significant trends were observed for the RLeg (HYA-HOA: 1.33 ± 0.55°; p = 0.090) (HYA-SOA: 1.38 ± 0.60°; p = 0.106). No other PhA comparisons were statistically significant but followed the trend HYA>HOA≥SOA. Significant group effects were found for all ECW segments (p = 0.001-0.014) and ICW in the LArm (p = 0.010), RLeg (p = 0.005), and LLeg (p < 0.001). No ECW or ICW comparisons reached statistical significance, but leg segments showed a ~ 0.24 L (ECW) and ~0.23 L (ICW) increase between groups (HYA<HOA<SOA).

**Conclusion**: Acute EAA intake had no significant effect on PhA, ECW, or ICW over time between HYA, HOA, and SOA. Significant group effects suggest raw BIA values are influenced by other factors. Although most pairwise comparisons were not statistically significant, the decrease in PhA with age and sarcopenia may indicate reduced muscle quality. The ECW increase between groups (HYA<HOA<SOA) may reflect edema and fluid accumulation, while the ICW increase requires further investigation.

**Acknowledgements**: DDC is on the advisory board for Shifted Supplements LCC. AAF and RRW are the inventors of the patented EAA supplement used in this study.

**Defining ‘REVERSE DIETING’: A Quantitative Survey of Post Diet Strategies Among Weight Loss Coaches for Natural Bodybuilding Athletes**

^a^Gretchen Shelton, ^a^Wayne A. Ayers-Creech, ^a^Samuel Blanke, ^a^Samuel Evers, ^a^Valentina Rodriguez, ^a^Talon Westervelt, ^a^Indira Alur,^a^Cody Logan, ^a^Vladyslava Martynovska, ^a^Cassandra Resler,^a^Bill Campbell

**^a^**Performance & Physique Enhancement Lab, Exercise Science Program, University of South Florida, Tampa, FL, USA

**Background**: The term ‘reverse-dieting’ is a popular strategy employed among coaches in physique-based sports such as bodybuilding. However, there’s no clear consensus of what constitutes a reverse diet in the literature. Coaches in this profession likely rely on anecdotal evidence when employing these protocols for their athletes. It is important to determine the current reverse-dieting practices among coaches to better establish evidence-based guidelines following a fat-loss period. Therefore, the primary purpose of this study is to define reverse dieting based on current professional practices utilizing a survey-based approach.

**Methods**: An anonymous online survey, collaboratively developed by the authors, was conducted using Qualtrics software (Qualtrics, Provo, UT, USA). The questions aimed to identify common coaching practices related to reverse diet implementation based on the coaches’ experience and clientele demographics. Statistical analysis involved preprocessing the dataset and computing descriptive statistics, including frequencies, percentages, means, and standard deviations using Python within the Anaconda Navigator v2.5.0, Jupyter Notebook integrated development environment v6.5.4.

**Results**: Results, reported as a percentage of total respondents, revealed a varied distribution of clientele populations with the lifestyle/general population comprising the largest proportion (82%), followed by competitive physique athletes (12%), strength athletes (4%), and team sports/athletics performance (2%). Most coaches had extensive experience, with 49% coaching for more than 5 years. Following a fat-loss phase, most coaches implemented a ‘reverse dieting’ approach (84%), focusing on increasing daily caloric intake. 65% of coaches preferred modifying macronutrient intakes as opposed to modifying general caloric intake (35%). On average, coaches increased daily caloric intake by 116.7 and 169.1 kcal increase per week (11.7% for females and 8.5% for males respectively), with an average duration of a reverse diet being 8.7 weeks. 74% of coaches reported decreasing cardio/step count post-fat loss phase.

**Conclusions**: Reverse dieting is the gradual increase of weekly caloric intake by approximately 8-12% over 9 weeks while reducing caloric expenditure to achieve energy balance. The findings of this study highlight reverse dieting protocols utilized by coaches after fat loss phases. The variation observed in macronutrient manipulation strategies reflects the nuanced approaches coaches employ in addressing individual client needs and preferences. Furthermore, the reported decrease in cardio/step count post-fat loss phase suggests a shift toward energy balance. These insights contribute to evidence-based coaching practices and highlight areas for further exploration, such as the efficacy of different post-diet protocols on weight loss maintenance.

**Caffeine effects on physical performance and sport-specific skills in elite youth soccer players: a randomized trial using the balanced placebo design**

Bezuglov Eduard^a,b^, Vakhidov Timur^a,b^, Achkasov Evgeniy^a^, Emanov Anton^b^, Koroleva Egana^b^, Kapralova Elizaveta^a^, Malyakin Georgiy^b^, Morgans Ryland^a^, Talibov Oleg^c^

^a^Department of Sports Medicine and Medical Rehabilitation, Sechenov First Moscow State Medical University, Moscow, Russia; ^b^High Performance Sports Laboratory, Sechenov First Moscow State Medical University, Moscow, Russia; ^c^Moscow State University of Medicine and Dentistry, Moscow, Russia

Corresponding Authors: Kapralova Elizaveta at kapralovaeliz@gmail.com

**Background**: There is a lack of studies on the effects of caffeine in relatively high doses on the performance and sports-specific skills of young soccer players conducted under stereotypical conditions.^1^ The main study objective was to examine the pharmacological and expected effect of acute caffeine ingestion (400 mg) on speed, strength, speed endurance, dribbling, and change of direction in elite young soccer players on a soccer field under stereotypical conditions.

**Methods**: 54 soccer players (age 15.93 ± 0.8 years, height – 180 ± 8.28 cm, weight – 69.45 ± 8.82 kg, BMI – 21.36 ± 1.37 kg/m2, somatic maturation degree – 98.05 ± 1.90), from a leading Russian soccer academy took part in a randomized trial using the balanced placebo design.

They were divided into 4 groups: 1 – told caffeine/given caffeine, 2 – told caffeine/given placebo, 3 – told placebo/given placebo, 4 – told placebo/given caffeine. All participants consumed two identical capsules 60 minutes before testing, each containing 200 mg of caffeine or placebo.

Physical performance and sport-specific skills were assessed using: 5, 10, 20 and 30 meter sprint, counter-movement jump, change of direction, dribbling, T-test and Repeated Sprint Ability (RSA) test. The incidence of side effects was assessed 24 hours after caffeine consumption using a questionnaire.^2^

**Results**: The data obtained showed that a single caffeine dose of 400 mg 60 minutes prior to the activity had a positive effect in groups 1 and 4 on such parameters of the RSA test such as fatigue index (p < 0.001) and the percentage decrement score (p < 0.001). No such effects were observed in groups 2 and 3. In group 1, there was a statistically significant improvement in dribbling performance (p < 0.048), while in group 4 there was only a tendency (p < 0.064). At the same time, caffeine had no effect on sprint time, change of direction, or jump height. The caffeine regimen was also shown to be safe – there was no statistically significant difference in the incidence of side effects between the groups (p = 0.56). No influence of caffeine expectancies on performance was observed.

**Conclusion**: The acute caffeine ingestion of 400 mg can be considered justified and safe in young soccer players aged 15-17 years with a high degree of somatic maturation.

**Keywords**: caffeine, young athletes, repeat sprint ability, soccer, sprint, strength, side-effects

References[1]Guest
NS, VanDusseldorp
TA, Nelson
MT, Grgic
J, Schoenfeld
BJ, Jenkins
NDM, Arent
SM, Antonio
J, Stout
JR, Trexler
ET, Smith-Ryan
AE, Goldstein
ER, Kalman
DS, Campbell
BI.
International society of sports nutrition position stand: caffeine and exercise performance. J Int Soc Sports Nutr. 2021;18(1):133388079
10.1186/s12970-020-00383-4PMC7777221[2]Muñoz
A, López-Samanes
Á, Aguilar-Navarro
M, Varillas-Delgado
D, Rivilla-García
J, Moreno-Pérez
V, Del Coso
J.
Effects of CYP1A2 and ADORA2A genotypes on the ergogenic response to caffeine in professional handball players. Genes (Basel). 2020;11(8):933.32823594
10.3390/genes11080933PMC7464361

**Effects of acute calcium ascorbate and ascorbic acid ingestion on pharmacokinetic profiles and markers of immunity in adult women and men**

Broderick L. Dickerson^a^, Drew E. Gonzalez^a^, Ryan J. Sowinski^a^, Dante Xing^a^, Megan Leonard^a^, Jacob Kendra^a^, Victoria Jenkins^a^, Choongsung Yoo^a^, Joungbo Ko^a^, Syamkumar Sivasankara Pillai^b^, Jigna Rajeshkumar Bhamore^b^, Bhimanagouda S. Patil^b^, Gus A. Wright^c^, Christopher J. Rasmussen^a^, Richard B. Kreider, FISSN^a^

^a^Exercise & Sport Nutrition Lab, Texas A&M University, College Station, TX 77843, USA; ^b^Vegetable & Fruit Improvement Center, Texas A&M University, College Station, TX USA; ^c^Flow Cytometry Facility, Texas A&M University, College Station, TX, 77843-4467 USA

Corresponding Authors: rbkreider@tamu.edu

**Background**: Comparator clinical trials with calcium ascorbate (CA), relative to ascorbic acid (AA), have shown higher intracellular leukocyte vitamin C concentrations for up to 24 hours following acute doses of 1,000 mg. This study evaluated whether lower doses of AA or CA promote differential effects on vitamin C pharmacokinetics over 32 hours, whole blood cell counts, neutrophil phagocytic function, and/or lymphocyte differentiation.

**Methods**: Two separate double-blind, randomized, and crossover manner studies were conducted to assess low (250 mg, *n* = 46) and high (500 mg, *n* = 47) acute single doses of AA or CA (Ester-C®, The Bountiful Co., Ronkonkoma, NY, USA) on plasma and lymphocyte ascorbic acid (ASC), dehydroascorbic acid (DHA), and total vitamin C levels, pharmacokinetic profiles (PK), cell blood counts (CBC), neutrophil phagocytic function, and lymphocyte differentiation. Participants were randomly assigned to low- or high-dose studies and AA or CA treatments. Fasted whole blood, plasma, serum, buffy coat, and peripheral blood mononuclear cell (PBMC) blood samples were collected before ingestion of the assigned supplement and after 1, 2, 4, 8, 24, and 32 hours. Participants were given three low-vitamin C-containing meals at designated times after the PK test to standardize nutrient intake. Data were analyzed using a general linear model (GLM) multivariate and univariate analysis with repeated measures, pairwise comparisons, and mean changes from baseline with 95% confidence intervals (CI). Data are presented as mean [UL, LL] CIs.

**Results**: Although some time effects were seen in the low-dose study, ingestion of 250 mg of CA did not promote significantly different effects than AA on plasma or lymphocyte ASC, DHA, total vitamin C, PK profiles, CBC, or neutrophil function, with the exception that 32-h lymphocyte total vitamin C tended to be higher with CA (5.0 μg/mL [−0.3, 10.3], *p* = 0.066). Analysis of the 500 mg dose results revealed time (*p* < 0.001, η_p_^2^ = 0.447, large effect) and treatment x time effects (*p* < 0.001, η_p_^2^ = 0.031, small effect) for plasma levels of ASC, DHA, and total vitamin C. Analysis of mean changes from baseline revealed that more ASC was oxidized to DHA levels with CA ingestion, resulting in a significantly greater (p < 0.001) area under the curve in DHA, potentially serving as a better source of vitamin C as an antioxidant to the brain and other tissues that absorb DHA more effectively. GLM analysis of lymphocyte concentrations of ASC, DHA, and total vitamin C revealed a time (*p* < 0.001, η_p_^2^ = 0.156, large effect) and treatment x time effect (*p* < 0.001, η_p_^2^ = 0.034, small effect). Analysis of mean changes from baseline revealed that ASC, DHA, and total vitamin C levels increased above baseline at most data points. PK analysis revealed that ASC distribution/absorption slope (−0.026 1/hr [−0.052, 0.001], *p* = 0.058) and rate (0.059 1/hr [−0.002, 0.121], *p* = 0.058) tended to differ between treatments. No significant differences were observed between treatments in DHA PK parameters. Total vitamin C volume distribution (Vd) (−94,999 ml [−180,784, −9216], *p* = 0.030) was significantly lower, while Vd area (−29,743 ml [−62,002, 2,517] *p* = 0.070) and relative Vd area (−427 ml/kg [222, −869], *p* = 0.058) tended to be lower and clearance (CL) observed area (13.2 ml/hr [−1.2, 27.7], *p* = 0.073) and CL exponential (3,251 ml/hr [−116, 6.617], *p* = 0.058) tended to be higher with CA. Immune marker analysis revealed that CA ingestion tended to increase neutrophils (*p* = 0.071) and reduce lymphocytes(*p* = 0.093) to a greater degree after 4 hours of ingestion. Neutrophil functionality analysis revealed that the percentage of neutrophils with (*p* = 0.070, η_p_^2^ = 0.038, small effect) and without phagocytosed bacteria (*p* = 0.068, η_p_^2^ = 0.038, small effect) tended to interact with the percentage of neutrophils containing phagocytosed bacteria in the CA group increased significantly from baseline (6.78 % [0.8, 12.8], *p* = 0.027) while not significantly affected by AA ingestion. Finally, lymphocyte cell differentiation analysis revealed a time effect (*p* < 0.001, η_p_^2^ = 0.707, large effect) with no treatment x time effect (*p* = 0.457, η_p_^2^ = 0.217, large effect). Pairwise comparison analysis revealed that CD25+ & CD127+ | Regulatory T cells tended to be lower after 24 hours with CA treatment (−6.09 [12.8, 0.6], *p* = 0.072), while CD16+ & CD56+ |Natural Killer Cells were significantly higher after 24-hours with CA (12.2 [2.7, 21.7], *p* = 0.012).

**Conclusions**: Ingestion of 250 mg of CA does not appear to promote greater benefits than AA. However, evidence showed that ingesting 500 mg of CA promoted greater conversion of ASC to DHA, volume distribution, and clearance while increasing neutrophil function and promoting a greater increase in CD16+ & CD56+ |Natural Killer Cells. These findings support claims that ingesting 500 mg of CA can affect immune markers and function for up to 24 hours and is a more effective source of vitamin C than AA.

**Acknowledgments**: This study was funded by a service contract from The Bountiful Co. (Ronkonkoma, NY, USA) and Nestlé Health Science USA (Bridgewater, NJ).

**Internal and external workload during an atypical women’s collegiate volleyball season**

Alexa J. Chandler^a^, Harry P. Cintineo^b^, Bridget A. McFadden^c^, Caroline S. Vincenty^a^, Mallory P. Dixon^a^, Gabriella Hickman^a^, Gianna F. Mastrofini^a^, Blaine S. Lints^a^, Sten Stray-Gundersen^a^, Shawn M. Arent, FISSN^a^

^a^Department of Exercise Science, University of South Carolina, Columbia, SC, USA; ^b^Department of Kinesiology, Lindenwood University, Saint Charles, MO, USA;^c^Department of Family, Nutrition, and Exercise Science, Queens College, City University of New York, Flushing, NY, USA

**Background**: The National Collegiate Athletics Association (NCAA) women’s indoor volleyball season typically occurs in the fall. However, during the 2020-2021 season, competitions spanned both fall and spring semesters for the NCAA Southeastern Conference (SEC). Given the unprecedented nature of this season format, the purpose of this study was to assess internal and external workloads across different phases of a year-long collegiate volleyball season.

**Methods**: NCAA SEC Division I female indoor volleyball athletes (N = 18; age = 19.9 ± 1.4 y) were observed during the 2020-2021 season. Microsensor technology (Polar TeamPro, Woodbury, NY, USA) was used to measure workloads during all team activities. Internal workloads were determined via heart rate (HR) using Edwards TRIMP, and external workload metrics were assessed via accelerometry and included total distance covered, distance covered in speed zones 4 (>15 – 19 km·h^−1^) and 5 (>19 km·h^−1^), and number of accelerations (>2.0 m·s^−2^). The season was separated into three segments: preseason (10 days), fall (64 days), and spring (57 days). Data were compiled using a 7-day rolling average and linear mixed effects models were used to determine differences between segments while accounting for number of days within each segment. Post-hoc tests with Tukey’s adjustment were conducted to determine differences between segments when significant main effects were present. An alpha level of 0.05 was used to determine statistical significance.

**Results**: Internal workload was highest during preseason (P < 0.0001) and was higher during spring compared to fall (P = 0.017). Total distance covered, distance in speed zone 4, and number of accelerations were highest during preseason (P < 0.0001), but there were no differences between fall and spring (P > 0.05). Further, there were no differences between any segments for distance in speed zone 5 (P = 0.098).

**Conclusions**: The current analysis found the highest internal and external workloads occur during the preseason, which is consistent with previous data from NCAA athletes. Interestingly, in this year-long analysis, internal workloads were higher in the spring compared to the fall, but there were no differences in external workload between semesters. The differential patterns between internal and external workloads may point to physiological responses to accumulated fatigue throughout a year-long season. Importantly, these responses may be exacerbated during a typical season, as fatigue may accumulate faster due the condensed schedules for NCAA sports. Assessing both internal and external workloads provides practitioners with a comprehensive understanding of stressors incurred by athletes as an uncoupling of the two metrics may provide insight on recovery and readiness.

**Acknowledgments**: This project was made possible in part by Grant Number T32-GM081740 from NIH-NIGMS award to GFM. Its contents are solely the responsibility of the authors and do not necessarily represent the official views of the NIGMS or NIH.

**Effects of Supplementation with Microalgae containing Fucoxanthin on Cognitive Function in Healthy and Active Late Middle-Aged Individuals Experiencing Initial Signs of Memory and Cognitive Decline III: Psychological Assessment**

Sarah Johnson^a^, Choongsung Yoo^a^, Joungbo Ko^a^, Dante Xing^a^, Jisun Chun^a^, Drew E. Gonzalez^a^, Victoria Jenkins^a^, Broderick Dickerson^a^, Megan Leonard^a^, Landry Estes^a^, Jacob Broeckel^a^, Jonathan Maury^b^, Rémi Pradelles^b^, Ryan Sowinski^a^, Christopher J. Rasmussen^a^, Richard B. Kreider, FISSN^a^

^a^Exercise & Sport Nutrition Lab, Texas A&M University, College Station, TX 77843, USA; ^b^Research & Development Department, Microphyt, 34670 Baillargues, FRA

Corresponding Authors: rbkreider@tamu.edu

**Background**: An emerging area of active and sports nutrition is helping people maintain physical performance and cognitive function as they age. We reported that supplementation with the microalgae extracts Phaeodactylum tricornutum (PT) containing the carotenoid fucoxanthin (4.4 and 8.8 mg/d) enhanced measures of cognitive function in young adult e-gamers. This study examined whether supplementation with PT containing 8.8 mg/d of fucoxanthin affects cognitive function in healthy, late middle-aged, physically active individuals experiencing self-perceived memory and cognitive decline.

**Methods**: 43 healthy late-middle-aged females and males (64.3 ± 6 years, 79.8 ± 16 kg, 27.0 ± 4 BMI) with non-clinical self-perceptions of memory and cognitive decline and a Mini-Mental State Examination (MMSE) score >24 (28.8 ± 0.9) participated in a randomized, double-blind, placebo-controlled clinical trial. Participants were familiarized with and practiced all cognitive tests to establish reliability. After baseline testing, participants were matched on sex and BMI and randomly assigned to supplement their diet with a placebo (PL) or 1100 mg/day of PT standardized for 0.8% (8.8 mg) fucoxanthin (FX, Brainphyt™, Microphyt, Baillargues, FRA) for 12 weeks while maintaining their regular diet and physical activity levels. Fasting blood samples and cognitive tests were performed at 0, 4, and 12 weeks of intervention. Data were analyzed using a general linear model (GLM) multivariate and univariate analysis with repeated measures with pairwise comparisons and mean changes from baseline with 95% confidence intervals (CI). Categorical data were analyzed by Chi-squared analysis. Differences with *p* < 0.05 were considered statistically significant, while differences with *p* > 0.05 to *p < *0.10 with medium (0.06) to large (>0.14) partial eta squared (η_p_^2^) effect sizes considered statistical tendencies. Means and 95% CI’s completely above or below baseline were considered clinically significant. Data are presented as mean [UL, LL] CIs with observed p-levels.

**Results**: Overall GLM analysis revealed no significant time (*p* = 0.151, η_p_^2^ = 0.101, medium effect) or treatment x time effects (*p* = 0.943, η_p_^2^ = 0.033, small effect) for the Profile of Mood States (POMS) 65-item questionnaire results. Analysis of mean changes from baseline revealed that the PL group had lower tension (Week 4: −1.667 [−3.389, 0.056], *p* = 0.057), depression (Week 4: −1.762 [−3.644, 0.120], *p* = 0.066), fatigue (Week 4: −2.476 [−4.021, −0.931], *p* = 0.002), confusion (Week 4: −1.190 [−2.173, −0.208], *p* = 0.019; Week 12: −1.286 [−2.399, −0.173], *p* = 0.025), and total mood disturbance (Week 4: −5.810 [−14.925, 3.306], *p* = 0.004) scores than the FX group. The FX group expressed higher vigor mean change from baseline scores (Week 4: 1.545 [−0.174, 3.265], *p* = 0.037) than the PL group. In terms of the Leeds Sleep Evaluation results, overall GLM analysis revealed no significant time (*p* = 0.155, η_p_^2^ = 0.157, large effect) or treatment x time effects (*p* = 0.109, η_p_^2^ = 0.166, large effect). Analysis of mean changes from baseline revealed the PL group had higher ratings for the question ‘How would you describe the way you currently fall asleep in comparison to usual? [More difficult than usual – Easier than usual]’, (Week 4: 7.905 [0.677, 15.132], *p* = 0.033) than the FX group. The FX group expressed higher ratings for the following questions: ‘How would you describe the quality of your sleep compared to normal sleep? [More restless than usual – Calmer than usual]’, (Week 4: 6.682 [−1.164, 14.528], *p* = 0.093); ‘How would you describe your awakening in comparison to usual? [More difficult than usual – Easier than usual]’, (Week 4: 7.682 [−0.811, 16.175], *p* = 0.075); ‘How would you describe your awakening in comparison to usual? [Requires a period of time longer than usual – Shorter than usual]’, (Week 4: 8.500 [0.756, 16.244], *p* = 0.032; Week 12: 6.636 [−0.988, 14.261], *p* = 0.086). Lastly, the Chi-squared analysis revealed statistical tendencies or differences between groups in the perceived stress responses for the following questions: ‘In the last month, how often have you felt nervous and “stressed”? (*p* = 0.084)’ ‘In the last month, how often have you found that you could not cope with all the things that you had to do?’ ‘In the last month, how often have you been able to control irritations in your life? (*p* = 0.085)’ and ‘In the last month, how often have you been angered because of things that were outside of your control? (*p* = 0.042).

**Conclusions**: These findings provide evidence that 1100 mg of PT, containing 8.8 mg of FX, improved subjective measures of sleep and perceived stress in late middle-aged individuals maintaining regular diet and physical activity patterns who perceive some memory and cognition; however, there were no favorable impacts on the POMS questionnaire responses for the FX group.

**Acknowledgments**

This study was funded by a grant from Microphyt, Baillargues, France.

**Examination of 14-day creatine monohydrate supplementation strategies on body composition and water distribution in female recreational athletes**

Isaac H. Avon, Kyle S. Levers, Natalia Wasilcyzk, Eden Glick, Eleanor U. Flacke, Anneliese Silverman, Payton Lynch, Alex Rainey, Henry Ball, Ashleigh Sorokin, Andrew M. Stranieri, Yichen Jin, Todd H. Miller

Department of Exercise & Nutrition Sciences, George Washington University, Washington, DC, USA

Corresponding Authors: klevers@gwu.edu

**Background**: Creatine monohydrate (CM) supplementation has been reported to increase cellular water retention and alter body composition in anaerobically-training cohorts, but the research in female athletes is limited. The study purpose was to examine the impact of two 14-d CM supplementation strategies on body composition and water distribution in free-living female recreational athletes.

**Methods**: 11 college-aged female recreational athletes (Mean ± SD: 19.90 ± 1.51 yrs, 163.88 ± 7.05 cm, 64.83 ± 9.43 kg, 29.55 ± 6.03% BF) participated in 3 testing sessions across the 14-day supplementation timeframe: 0-day (0D), 7-day (7D), and 14-day (14D). Participating subjects were actively training 4-8 hours/wk, consuming <2 g/d of dietary creatine, and free of creatine supplementation >2 mo. Subjects were randomly assigned to 3 supplement groups in a double blind manner: loading (CF-L, 20 g CM + 15 g Maltodextrin), maintenance (CF-M, 5 g CM + 30 g Maltodextrin), and placebo (PLA, 35 g Maltodextrin). Subjects ingested the supplement 1x/d before noon and maintained standardized conditions (fasted (>6hrs), caffeine-free (>12hrs), exercise (>24 hrs) before testing with study period alcohol abstinence. Body composition and water distribution were assessed via dual-energy X-Ray absorptiometry (DXA) and multi-frequency bioelectrical impedance analysis (BIA), respectively, with pretest urine specific gravity hydration confirmation. Two-way repeated measures ANOVAs with Tukey HSD post hoc testing evaluated specific outcome metrics across groups and time.

**Results**: Despite vastly different 14-d CM supplementation strategies, body weight (BW, *p* = 0.109, ηp^2^ = 0.744), body fat % (BFp, *p* = 0.493, ηp^2^ = 0.162), fat mass (FM, *p* = 0.411, ηp^2^ = 0.120), lean mass (LM, *p* = 0.833, ηp^2^ = 0.045), and fat-free mass (FFM, *p* = 0.844, ηp^2^ = 0.042) did not change. Similarly, intracellular water (ICW, *p* = 0.189, ηp^2^ = 0.471), extracellular water (ECW, *p* = 0.170, ηp^2^ = 0.530), and total body water (TBW, *p* = 0.181, ηp^2^ = 0.496) remained uneffected, while ECW/TBW demonstrated 0D-14D modification (*p* = 0.004, ηp^2^ = 0.595). CF-L decreased ECW/TBW (Δ-1.35%, *p* = 0.004) 0D-14D, while CF-M (Δ-0.45%, *p* = 0.939) and PLA (Δ0.74%, *p* = 0.451) facilitated insignificant ratio alteration.

**Conclusion**: 14-d creatine supplementation in female recreational athletes during an active training period did not facilitate significant tissue changes. However, 14-d CM loading (CM-L) lowered ECW/TCW, perhaps facilitating greater intracellular water and nutrient retention compared to maintenance (CM-M) strategies. Aesthetic or weight class-based female athlete populations may derive metabolic and neuromuscular benefits from moderate duration CM loading without significant tissue accumulation.

**Acknowledgements**: This research was partially supported by Live Momentous (Park City, UT, USA). This supporter played no role in data acquisition, data management, nor data interpretation.

**Defining ‘reverse dieting’: A qualitative survey of post diet strategies among weight loss coaches for natural athletes**

^a^Gretchen Shelton, ^a^Wayne A. Ayers-Creech, ^a^Derong Wu, ^a^Sydney Monahan, ^a^Cassidy Bale, ^a^Grace Chong, ^a^Landon Shannahan, ^a^John Solis, ^a^Sara Hobbes, ^a^Dionellie Batista-Ramirez, ^a^Bill Campbell

**^a^**Performance & Physique Enhancement Lab, Exercise Science Program, University of South Florida, Tampa, FL, USA

**Background**: Reverse dieting is a post-diet strategy growing in popularity in the fitness space. The present study sought to better understand fitness professionals’ rationale for utilizing reverse diets for their clients. Research on this topic is limited and not operationally defined in the literature.

**Methods**: The online survey was developed collaboratively by the authors and conducted through Qualtrics Software (Qualtrics, Provo, UT, USA). This study aimed to target coaches of drug-free athletes who utilize what they consider reverse dieting. This study primarily focused on the two open-ended questions in the survey: ‘Why do you implement a reverse dieting strategy after a caloric deficit (fat loss phase) as opposed to another approach?’ and ‘If your clients have ever experienced a negative effect from reverse dieting explain. (E.g. adherence, body fat levels, hormonal factors, etc.)’. The qualitative nature of the data was then sorted through a content analysis approach to identify keywords into overarching categories. Subcategories were then identified if appropriate to promote further discussion on the topic.

**Results**: This survey collected 66 responses for two open-ended questions. When asked, ‘Why do you implement a reverse dieting strategy after a caloric deficit (fat loss phase) as opposed to another approach?’, 47 out of 66 respondents stated it was for the benefits in developing sustainable eating habits. 31 respondents mentioned metabolic adaptations and 20 respondents alluded to physiological health considerations. 4 out of 66 statements were identified as other/uncategorized statements, as they did not fit into the predefined categories.

The second question posed ‘If your clients have ever experienced a negative effect from reverse dieting explain (e.g. adherence, body fat levels, hormonal factors, etc.)’. 27 out of 66 respondents indicated that they had ceased the reverse diet due to adherence issues, 20 responses indicated no side effects from reverse dieting, 10 responses mentioned increased body fat levels, and 4 pointed toward hormonal factors. 7 responses were categorized as ‘Other/Uncategorized’ due to insufficient detail, however psychological effects, body image, hunger, digestive issues and emotional reasons were mentioned.

**Conclusion**: The results of this survey offer valuable insight and anecdotal experience regarding utilization and cessation of reverse dieting among fitness professionals. The most notable finding is that reverse dieting is often employed to promote sustainable eating habits, while the most common reason for discontinuation is a lack of adherence. These results collectively highlight a paradox in the implementation of reverse diets, suggesting their application is highly interindividual.

**Protein yogurt consumption results in similar body composition and strength gains but differential effects on gut microbiome than whey protein in untrained older adults after 8 weeks of strength training: A randomized trial**

Matías Monsalves-Álvarez^a,g^*, Marcelo Flores-Opazo^b^, Thomas Haynes^c^,Sofia Badilla^c^, Francisco Sánchez^c^, Claudio Pérez de Tudela^c^, Yildy Utreras-Mendoza^d^, Cristian Campos^a^, Álvaro Reyes^c^, José Gomez^c^, Badir Zara^c^, Carlos Sepúlveda^d^, Rodrigo Troncoso^e^, María Paulina Correa^d,g^, Paulina Calderón-Romero^f^, Felipe A.Court^f,g^ and Denisse Valladares-Ide^b^

^a^Exercise and Rehabilitation Sciences Institute, Faculty of Rehabilitation Sciences, Universidad Andrés Bello, Santiago, Chile,^b^ Institute of Health Sciences, Universidad de O´Higgins, Rancagua, ^c^Chile Motion Health and Performance Center, Lo Barnechea, Chile, ^d^ Centro de Fisiología Celular e Integrativa, Facultad de Medicina, Universidad del Desarrollo, ^e^ Laboratorio de Investigación en Nutrición y Actividad Física, Instituto de Nutrición y Tecnología de los Alimentos, Universidad de Chile, Santiago, Chile, ^f^ Center for Integrative Biology, Faculty of Sciences, Universidad Mayor, Santiago, Chile, ^g^ Geroscience Center for Brain Health and Metabolism (GERO), Santiago, Chile.

*Corresponding Authors: matias.monsalves@unab.cl

**Background**: Proteins are essential nutrients to improve muscle health during aging. Amongst different animal sources, dairy proteins have great potential due to their micronutrient content, bacteria cultures, and complete amino acid profile, particularly leucine. Dairy proteins also contain casein and whey, prime sources of protein supplements. These include caseinates, concentrates, isolates, and hydrolyzed proteins, each with different absorption speed, digestibility, and muscle protein response. Although the effects of combining resistance training with whey protein supplementation on muscle mass/strength gains are widely accepted, evidence of these benefits from other protein sources, such as yogurt, is scarce, especially in older adults. This study aimed to compare the effects of daily consumption of a high-protein yogurt or whey protein isolate in combination with supervised RT for eight weeks on body composition, muscle mass/strength gains, functional performance, and microbiome changes in older adults.

**Methods**: This randomized trial (NCT06412302) recruited 18 untrained older adults (65.8 ± 4.1y) who undertook 3 weekly sessions of supervised RT for 8 weeks. Half of the participants were randomly assigned to receive either 25 g of Whey protein (PY) (ISO100, Dymatize, USA) or 24.5 g of protein from high-protein yogurt (YP) (Loncoleche, Chile) immediately after each session. RT sessions consisted of 3 × 8-12 reps, 70-85% of 10RM at 1-2 repetitions in reserve. All sessions were supervised by a certified trainer. Loads were adjusted every 2 weeks to maintain exercise intensity during the protocol. Assessments of body composition by bioelectric impedance, 10-RM, resting energy expenditure, 24h-recall food intake, VO_2peak_, time-up & go [TUG], hand-grip strength [HGS], and isokinetic strength were performed before and at the end of the trial. Additionally, whole-blood and stool samples for 16S rRNA gene sequencing were collected.

**Results**: 17 subjects finished the 8-week protocol WP (n = 8) and YP (n = 9). WP decreased fat mass (%) compared with PY (WP: Pre:33.9 ± 5.1%, Post: 32.4 ± 5.0%, p = 0.00; PY: Pre:31.66 ± 7.4%, Post: 30.8 ± 7.0%, p = 0.115). Skeletal muscle mass was significantly increased in PY (Pre:24.3 ± 6.3 kg, Post: 24.8 ± 6.5 kg, p = 0.04; WP: Pre:26.3 ± 8.9 kg, Post: 26.6 ± 8.9 kg, p = 0.07). VO_2peak_ and RMR tended to improve in PY compared with WP, p = 0.054 and p = 0.07, respectively. Muscle strength gains in leg press, leg curl, leg extension, pull-down, and bench press were obtained with both interventions (Pre vs Post; p = 0.001) with no differences between groups. Dominant leg (Right) isokinetic also improved in both groups (PY: Pre:54.9 ± 40.7 Nm, Post: 64.2 ± 49.5 Nm, p = 0.00; WP: Pre:63.8 ± 53.6 Nm, Post: 74.5 ± 61.9 Nm, p = 0.00). Similarly, TUG decreased significantly in both groups (WP: 5.7s to 5.0s, PY: 5.3s to 4.7s; p = 0.00 and p = 0.02). Microbiome sequencing analysis in stool samples revealed differential changes in the genus in WP with significant increases in DTU089 (p = 0.01), *Marvinbryantia* (p = 0.04), and *Ruminococcaceae* incertae sedis (p = 0.02) and decrease in *Prevotella* (p = 0.02). The PY group showed an increase in *Coprococcus* (p = 0.01), *Oscillibacter* (p = 0.02), *Parasutterella*(p = 0.03) and reduction in *Hungatella* (p = 0.04). Regarding Phyllum, only WP results in a significant change in Firmicuntes (p = 0.04) compared with PY, while biodiversity determined by the Shannon Entropy analysis showed that PY induced a significant increase after intervention (p = 0.04)

**Conclusions**: Daily intake of 25 g of protein from yogurt or a whey protein isolate during RT induced similar benefits in body composition, physical function, and strength gains but affected the microbiome genus, phylum, and diversity differentially. This difference could be associated with protein digestibility or amino acid composition, which deserves further research to elucidate the role of different protein sources in gut health.

**Fundings**: This work was supported by the Consorcio Lechero Award, Sociedad Chilena de Nutrición and Fondecyt Iniciación (N°11230186): MMA and (ANID-ACT210006):MPC and Geroscience Center for Brain Health and Metabolism, FONDAP-15150012: PCR and FC.

**The Effects of a High-Carbohydrate, High-Glycemic Load Hypocaloric Diet on Body Fat Loss – A Case Study**

John Solis^a^, Cassidy Bale^a^, Kworweinski Lafontant^a,b^, Wayne A. Ayers-Creech^a^, Gretchen Shelton^a^, Alexa Rukstela^a^, Yasamian Alsayed^a^, Yuto Ohigashi^a^, Indira Alur^a^, Cassandra Resler^a^, Jordan Moon^c^, Samuel Buckner^a^, Mike Matthews^d^, Bill Campbell^a^

^a^Performance & Physique Enhancement Lab, Exercise Science Program, University of South Florida, Tampa, FL, USA; ^b^Physiology of Work and Exercise Response (POWER) Lab, Institute of Exercise Physiology and Rehabilitation Science, University of Central Florida, Orlando, FL, USA; ^c^Body Quantification, LLP, Columbus, OH, USA, ^d^Legion Athletics, Inc., Clearwater, FL, USA

Corresponding Authors: bcampbell@usf.edu

**Background**: The carbohydrate-insulin model (CIM) posits that obesity is caused by excess consumption of carbohydrates (CHO), which then disrupts normal insulin metabolism, leading to weight gain. Some have taken the etiological and theoretical basis of CIM and deviated from it to assert that high-carbohydrate diets (even when consumed within an energy deficit) are not effective for losing body fat. The purpose of this case study was to challenge this application by exploring a high carbohydrate, high glycemic load hypocaloric diet to induce fat loss in a resistance-trained obese male.

**Methods**: The dietary protocol targeted a 25% caloric deficit, with approximately 60% of calories coming from carbohydrates (CHO) and 20% coming from protein (PRO) and fats equally. Refeeds and diet breaks were accounted for in the targeted deficit. The exercise program consisted of whole-body resistance training (3x/week) and aerobic exercise (3x/week). Body composition (4-compartment model) and select blood markers predictive of metabolic dysfunction (fasting glucose, fasting insulin, and hemoglobin A1C) were assessed at the beginning and end of the 30-week intervention. Data is presented descriptively.

**Results**: Average values for glycemic index (GI) and glycemic load during the study were 54 and 36, respectively. The average daily caloric intake was approximately 2,400 kcals, equating to a caloric deficit of ~22% per day. The macronutrient composition of the diet was 58% CHO, 14% PRO (0.83 g/kg/day), and 28% fats. Body weight was reduced by 11% (from 104.8 to 93.2 kgs) and losses were solely from fat stores. Fat mass declined from 31.1 to 18.9 kgs (−12.2 kgs), which was equivalent to a 9.4% decrease in body fat (from 29.7 to 20.3%). Fat-free mass was maintained during the study (73.6 to 74.3 kgs). Fasting glucose decreased from 95 to 91 mg/dL, fasting insulin decreased from 6.9 to 5.2 uIU/mL, and hemoglobin A1C slightly increased from 5.2 to 5.4%.

**Conclusions**: This study indicates that a carbohydrate-rich, high glycemic load hypocaloric diet is a viable strategy for losing body fat in an obese male and challenges the notion that body fat cannot be reduced while consuming a high carbohydrate/high glycemic load diet. Future discourse and application on dieting for weight loss should focus on a well-structured nutritional plan that employs a sustainable caloric deficit rather than focusing on one specific macronutrient or food group.

**Disclosures**. The authors report no conflicts of interest. Funding for blood work was provided by Legion Athletics, Inc.

**Relationship Between Performance Testing and Body Composition Following Off-Season Training in Division I Football Players**

Emma E. Worley^a^, Sten O. Stray-Gundersen^a^, Alexa J. Chandler^a^, Gianna F. Mastrofini^a^, Blaine S. Lints^a^, Shawn. M Arent, FISSN^a^

^a^Arnold School of Public Health, Dept. of Exercise Science, University of South Carolina, Columbia, SC.

**Background**: Performance tests and body composition measures are used to assess athlete status and monitor changes. Percent body fat (%BF) and fat-free mass (FFM) may be important contributors to certain aspects of athletic performance. However, less is known regarding relationships between changes in these metrics following off-season training. Therefore, we sought to determine the relationship between changes in body composition and performance following an 8-week off-season training program.

**Methods**: National Collegiate Athletic Association (NCAA) Division I football players (N = 106, age = 20.9 ± 1.7 y, height = 188.0 ± 5.9 cm, weight = 108.5 ± 21.2 kg) completed body composition and performance testing before and after eight weeks of winter off-season training. Body weight (BW) was measured using a scale to the nearest 0.1 kg, while FFM and %BF were calculated using a modified Jackson Pollock formula from ultrasound measures (BodyMetrix, Brentwood, CA, USA). Performance tests included the pro-agility-shuttle (PAS) (n = 80), 3-cone drill (n = 75), vertical jump (VJ) (n = 86), 102 kg bench press (BP) (n = 93), and 10-yard sprint (n = 95). Peak power (PP) was calculated by PP = (60.7) x (jump height [cm]) + 45.3 × (body mass [kg]) − 2055. Pre- to post- change scores (∆) were computed for each metric. Pearson-product moment correlations were calculated to quantify relationships between changes in each body composition and performance metric (α = 0.05).

**Results**: ∆PP was positively correlated with ∆%BF (r = 0.29, P = 0.006) and ∆BW was positively correlated with ∆VJ (r = 0.25, P = 0.022) while ∆FFM was positively correlated with ∆BP performance (r = 0.29, P = 0.005). However, there were no significant correlations between ∆FFM and ∆PAS, ∆10-yard sprint, ∆VJ, or ∆PP (P > 0.05). There were no significant correlations between ∆%BF and ∆PAS, ∆10-yard sprint, ∆VJ, or ∆BP (P > 0.05). However, ∆3-cone drill was positively correlated with ∆VJ (r = 0.25, P = 0.04), ∆10-yard sprint (r = 0.24, P = 0.04), and ∆PAS (r = 0.25, P = 0.04); while ∆BP was positively correlated with ∆PAS (r = 0.24, P = 0.04).

**Conclusion**: BW and %BF changes were positively associated with power changes while FFM changes were positively associated with upper-body strength changes following eight weeks of off-season training. However, due to individualized variation of BW and %BF, the correlation is weak. Factors outside of body composition may have greater influence on speed and change-of-direction performance, such as training for speed and power. As optimal body composition differs between positions, future research should elucidate any position-specific differences in relationships between body composition and performance.

**Acknowledgments**: This project was made possible in part by Grant Number T32-GM081740 from NIH-NIGMS award to GFM. Its contents are solely the responsibility of the authors and do not necessarily represent the official views of the NIGMS or NIH.

**Exploring The Relationships Between Low Energy Availability, Sports Nutrition Knowledge, and Resting Metabolic Rate in Recreational Women Athletes**

Meghan K. Magee^a^, Jennifer B. Fields^b^, Kathryn Byers^a^, Connor Franks^a^, Elise Gambol^a^, Thomas Lillard^a^, and Lindsay McPhail^a^

^a^Exercise Science and Exercise Physiology, Kent State University, Kent, OH, ^b^Department of Nutritional Sciences, University of Connecticut, Storrs, CT

**CONTACT**: mmagee3@kent.edu

**Background**: Low energy availability (LEA) occurs when an individual does not eat enough calories to keep up with the metabolic demands of their sport or training. LEA has been shown to negatively impact body composition and resting metabolic rate (RMR). Recreational athletes have been reported to suffer from LEA and may not realize due to a lack of resources and education.

**Purpose**: To explore relationships between LEA in females questionnaire (LEAF-Q), sports nutrition knowledge, and RMR in recreational women athletes.

**Methods**: 10 women (age: 22.4 ± 2.8 years; height: 164.3 ± 9.2 cm; body mass: 66.6 ± 13.0 kg) participated in this cross-sectional study. Participants arrived at the laboratory and underwent a bioelectrical impedance analysis (BIA). After completing BIA, participants were instructed to remain seated for 25 minutes while completing the LEAF-Q and the abridged sports nutrition knowledge questionnaire (ASNKQ). Once 25 minutes passed, participants were instructed to lie flat on a padded table for RMR assessment via a metabolic cart. RMR data was collected for 30 minutes. Bivariate correlations were run to evaluate the relationships between the following variables: LEAF-Q, ASNKQ, RMR (kcals), body fat percent (BF%), and fat free mass (kg; FFM). The following criteria were used for interpreting correlation coefficients: weak, 0.20–0.39; moderate, 0.40–0.59; strong, 0.60–0.79; and very strong, >0.80.

**Results**: Table 1 provides descriptive data and the relationships between each variable. ASNKQ had a very strong relationship with RMR and a strong relationship with FFM. FFM had a strong relationship with RMR and a moderate negative relationship with LEAF-Q. Finally, BF% had moderate relationships with ASNKQ and RMR.

**Table 1.** Descriptive and correlations.

Variable	Mean±SD	LEAF-Q	ASNKQ	RMR
LEAF-Q Score	6.5 ± 4.6			
ASNKQ Score	53.3 ± 13.3	0.158(0.663)		
Resting Metabolic Rate (kcals)	1594.8 ± 180.8	0.167(0.645)	0.827(0.003)***	
Body Fat Percentage	27.4 ± 5.0	−0.011(0.976)	0.501(0.141)*	0.559(0.093)*
Fat Free Mass (kg)	47.9 ± 7.2	−0.440(0.203)*	0.643(0.045)**	0.689(0.027)**

Data is represented as Pearson Correlation(p-values)RMR- resting metabolic rate; LEAF-Q- low energy availability in females questionnaire; ASKNQ- abridged sports nutrition knowledge questionnaire; SD- standard deviation*indicates a moderate relationship**indicates a strong relationship***indicates a very strong relationship

**Conclusion**: ASKNQ had a positive influence on FFM and RMR. LEAF-Q score had a moderate negative influence on FFM indicating higher LEAF-Q scores may lead to lower FFM. BF% had moderate positive relationships with ASKNQ score and RMR. It is possible this relationship has more relevant to total body weight.

**The effects of swearing on aerobic exercise and mood**

Alyana Andal, Alexandra Jimenez, Nick Washmuth, Jose Antonio FISSN, Lia Jiannine

Department of Health and Human Performance, Nova Southeastern University, Davie Florida

Corresponding Authors: Jose.Antonio@nova.edu

**Background**: The purpose of this investigation was to determine the impact of swearing on aerobic endurance and mood.

**Methods**: Fourteen individuals (male n = 5, female n = 9) participated in this randomized, counterbalanced, repeated measures study (mean ± SD, age = 21.6 ± 2.2 years, average hours of exercise training experience per week, 8 ± 4 hours). All participants reported to the laboratory twice to complete the Bruce protocol, with a minimum of 48 hours between sessions to minimize the potential effects of soreness. The Bruce protocol is a treadmill exercise test where speed and incline are incrementally increased every 3 minutes. Participants self-selected a swear word that they would use if they hit their head accidentally or stubbed their toe. Then, as a control, they were given a neutral word with similar sounds and syllables as their swear word. After participants were randomized to either an initial swearing or neutral word condition, participants completed the Profile of Mood States (POMS) questionnaire and then engaged in a 5-minute treadmill walking warm-up prior to completing the Bruce protocol. Participants were instructed to vocalize either their swear word or neutral word every 3 seconds for 30 seconds at the onset of each 3-minute stage during Bruce protocol, and to progress through the stages until exhaustion required them to stop. Upon reaching exhaustion, participants again completed the POMS.

**Results**: The results showed no significant difference in Bruce protocol times between the swearing and neutral word conditions (P > 0.05). Depression scores significantly decrease after swearing (P = 0.05). Fatigue and tension scores significantly increased after repeating the neutral word (P ≤ 0.05) and swearing prevented these negative changes.

**Conclusions**: These findings indicate that swearing may not impact aerobic endurance; however, swearing may decrease depression scores and prevent negative changes in mood scores.

**Glycoprotein matrix-bound iron improves iron concentrations compared to ferrous bisglycinate chelate and ferrous fumarate**

Jaci Kelly^a^, Nikolas Keratsopoulos^b^, Alyssa Faterkowski^a^, Katelyn Kolodziejczyk^a^, Mathis Rollin^a^, Robert Mills^a^, Mandy Parra^a^, Ariane Secrest^b^, Ralf Jäger^c^, Martin Purpua^c^, Grant M. Tinsley^d^, Lem W. Taylor^a,e^

^a^Human Performance Lab, School of Exercise & Sport Science, University of Mary Hardin-Baylor, Belton, TX, USA; ^b^Public Health Program, University of Mary Hardin-Baylor, Belton, TX, USA; ^c^Increnovo LLC, Whitefish Bay, WI, USA; ^d^Energy Balance & Body Composition Laboratory, Department of Kinesiology & Sport Management, Texas Tech University, Lubbock, TX 79409, USA; ^e^Doctor of Physical Therapy Program, University of Mary Hardin-Baylor, Belton, TX, USA

Corresponding Authors: ltaylor@umhb.edu

**Background**: Biotransformation of minerals via glycosylation by microorganisms such as yeast and/or probiotics yield nutrients bound to a food matrix resulting in increased bioavailability. The purpose of this study was to compare the absorption kinetics of a glycoprotein matrix-bound iron (GPM) compared to ferrous bisglycinate chelate (FBC) and ferrous fumarate (FF).

**Methods**: In a double-blind crossover design, 17 participants ingested 11 mg of iron as either GPM™ Iron (GPM, Ashland, Kearny, NJ, USA), FBC, or FF. Blood samples were taken at baseline, 30-, 60-, 90-, 120-, 180-, 240-, 300-, 360-, 420-, and 480-minutes post-ingestion. Linear mixed-effect models were used to evaluate the influence of condition and time on iron concentrations. Pharmacokinetic analysis was performed to establish the incremental area under the concentration vs. time curve (iAUC), maximum concentration (Cmax) and time of Cmax (Tmax) with a significance level of p < 0.05.

**Results**: Linear mixed-effect model terms indicated that there were statistically significant effects of the GPM condition, both for raw iron concentrations and changes from baseline (p = 0.03). On average, participants had iron concentrations that were 27.1 (95% CI: 2.8 to 51.4) mcg/dL higher after GPM consumption as compared to FF. Additionally, changes in iron concentrations from baseline were 16.6 (95% CI: 1.5 to 31.7) mcg/dL higher after GPM consumption as compared to FBC. In contrast, iron concentrations and iron changes after FBC consumption did not significantly differ from the reference condition (FF). Over the 8 hours following iron ingestion, GPM increased iron concentrations compared to FF and FBC by up to 35% and up to 33%, respectively. A significant effect of condition on iAUC was observed (p = 0.047), without statistically significant effects of condition for Cmax (p = 0.15), or Tmax (p = 0.81). Post hoc tests indicated a trend (p = 0.07) for a difference between iAUC in the GPM and FBC conditions, without differences between GPM and FF (p = 0.17) or FBC and FF (p = 0.75). For iAUC, the magnitude of the effect size for the GPM condition was ‘moderate’ as compared to FBC and FF. No side effects were reported.

**Conclusions**: These findings suggest that iron bound to a glycoprotein matrix can improve iron absorption, as indicated by concentrations over 8 hours post-ingestion and the iAUC, without significant differences observed in Cmax and Tmax, and without any associated side effects. These data could have implications for preventing anemia, strengthening the immune system, and promoting sleep quality and fetal health.

**Acknowledgements**: This study was funded by Ashland Inc. (Kearny, NJ). R.J. and M.P. are employed by Increnovo LLC and consultants to Ashland, the manufacturer of the material studied and sponsor of the study. R.J. and M.P. were not involved in collecting or analyzing the data. All other authors declare no conflicts of interest.

**Bioavailability of a beaded creatine delivery system on serum creatine levels and whole body creatine retention: proof of concept study**

Jisun Chun^a^, Joungbo Ko^a^, Choongsung Yoo^a^, Dante Xing^a^, Drew E. Gonzalez^a^, Broderick Dickerson^a^, Victoria Jenkins^a^, Megan Leonard^a,^ Kelly Hines^a^, Ralf Jäger, FISSN^b^, Martin Purpura^b^, Ryan Sowinski^a^, Christopher J. Rasmussen^a^, Richard B. Kreider, FISSN^a^

^a^Exercise & Sport Nutrition Lab, Texas A&M University, College Station, TX 77843, USA; ^b^Increnovo LLC, Whitefish Bay, WI, USA

Corresponding Authors: rbkreider@tamu.edu

**Background**: Creatine monohydrate (CrM) is the gold standard to compare the efficacy of creatine supplementation. The recommended loading dose is 4 × 5 g/d for 5 – 7 days because oral ingestion of CrM peaks in the blood in about one hour and returns to baseline in about 4 – 5 hours. Ingesting several 5 g doses of CrM throughout the day promotes rapid creatine retention. While CrM loading protocols are effective, there has been interest in determining ways to improve the pharmacokinetic (PK) profile and/or creatine retention. This study examined whether providing CrM in micro-beads or beadlets that theoretically would delay the release of creatine into the blood over time will affect PK profiles and/or whole-body creatine retention during the first 24 hours after ingestion compared to CrM.

**Methods**: Fifteen females and males (28 ± 6 years, 79.0 ± 14 kg, 26.5 ± 3 kg/m^2^) with no recent history of creatine supplementation participated in a double-blind, randomized, and crossover study with at least 7-days between testing sessions. Participants adhered to a plant-based protein diet prior to each testing session. Participants donated a pre-supplementation 24-hour urine sample and a fasting blood sample. Participants were then randomly assigned to ingest capsules containing 5 g of CrM or 5 g of CrM prepared in beadlets (CrM-Bead). Blood samples were collected at 0, 0.5, 1, 2, 4, 6, and 8 hrs. Participants then ingested another 5 g dose of the assigned supplement (total of 10 grams) and donated a 24-hour urine sample to assess whole-body creatine retention. Participants repeated the experiment after a seven-day washout while ingesting the remaining supplement. Serum and urine creatine content were determined using commercial ELISA kits. Whole-body creatine retention was determined by subtracting CrM intake from the difference between post-supplementation urine creatine content and expressed in grams and percent retention. Area under the curve values were calculated as the sum of delta value changes. Data were analyzed by GLM with repeated measures, pairwise comparisons, and mean changes from baseline with 95% confidence intervals (CIs). Differences with *p* < 0.05 were considered statistically significant, while differences with *p* > 0.05 *to p < *0.10 with medium (0.06) to large (>0.14) partial eta squared (η_p_^2^) effect sizes considered statistical tendencies. Means and 95% CI’s completely above or below baseline were considered clinically significant. Data are presented as mean [UL, LL] CIs with observed p-levels.

**Results**: Figure 1 shows that Ingestion of a 5-gram oral dose of CrM and CrM-bead significantly increased serum creatine levels (*p* < 0.001, η_p_^2^ = 0.858, large effect) with no interaction effects (*p* < 0.426, η_p_^2^ = 0.213, large effect). However, pairwise comparison analysis revealed that serum creatine levels tended to be lower after 1 hour of ingestion of CrM-Bead (−89 µM [−183, 7], *p* = 0.065) with no significant differences in AUC (CrM 586 ± 309, CrM-Bead 465 ± 292 µM, *p* = 0.28) suggesting a delayed release or greater uptake of creatine from the blood with CrM-Bead ingestion. Analysis of whole-body creatine retention revealed a similar percentage of 24-hour creatine retention (CrM 8.1 ± 1.14, CrM-Bead 8.36 ± 1.25 g, *p* = 0.76; CrM 81.8 ± 11.4, CrM-Bead 83.6 ± 12.5 %, *p* = 0.76).

**Figure 1.**
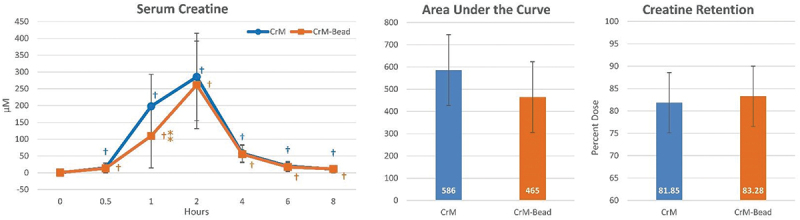
Serum creatine, the area under the curve, and whole-body creatine retention data. Data are means and ± 95% confidence intervals. ⁑ = *p* > 0.05 to *p* < 0.10 difference between treatments. † = *p* < 0.05 difference from baseline value.

**Conclusions**: This proof of concept study indicates that ingesting 5 grams of CrM-Bead can delay the release of CrM and affect pharmacokinetic profiles. While not significantly different, the percentage of creatine retention after 10 grams of CrM-Bead ingestion was slightly higher which could potentially affect total creatine retained during a traditional loading period. However, additional research is needed to evaluate the pharmacokinetics of CrM-Bead ingestion compared to CrM and whether using CrM-Bead as the source of creatine monohydrate in traditional loading and maintenance doses may influence muscle and other tissue retention.

**Acknowledgments**

This study was funded by Specnova, Inc. (Tysons Corner, VA, USA) in collaboration with Increnovo LLC (Whitefish Bay, WI, USA).

**Effects of chromium, *Phyllanthus emblica* fruit extract, and Shilajit supplementation on markers of cardiovascular health, fitness, and weight loss in men and women initiating an exercise and diet intervention program**

Victoria Martinez, Kay McAngus, Broderick Dickerson, Megan Leonard, Elena Chavez, Jisun Chun, Megan Lewis, Dante Xing, Drew E. Gonzalez, Choongsung Yoo, Joungbo Ko, Ryan Sowinski, Christopher J. Rasmussen, Richard B. Kreider, FISSN.

Exercise & Sport Nutrition Lab, Texas A&M University, College Station, TX 77843, USA

Corresponding Authors: rbkreider@tamu.edu

**Background**: Chromium (Cr) supplementation has been reported to improve insulin sensitivity. *Phyllanthus emblica* (PE) has been purported to minimize the conversion of Chromium III to Chromium VI, improve endothelial function, reduce platelet aggregation, and help manage blood glucose and lipid levels. Shilajit (SJ) has been reported to possess anti-inflammatory, adaptogenic, immunomodulatory, and anti-dyslipidemia properties. This study examined the effects of Cr, PE, and SJ supplementation on weight loss, body composition, and cardiovascular risk markers in sedentary and overweight men and women initiating a supervised exercise and weight loss program.

**Methods**: 65 men and women (49.7 ± 10 yrs., 34.2 ± 6 kg/m^2^, 39.6 ± 8 % fat) with at least 2 markers of metabolic syndrome participated in a randomized, placebo-controlled, parallel-arm, and repeated measures intervention trial. Volunteers participated in a standardized resistance training (3 sessions/week) and aerobic training (3 x 30 min sessions/week) program while reducing energy intake by 5 kcals/kg/d. In a double-blind and randomized manner, participants were matched by age, sex, BMI, and body mass to supplement their diet with placebo (PLA), 400 mcg of trivalent chromium with 6 mg of PE and 6 mg of SJ (Cr-400, Crominex®3^+^, New Brunswick, NJ, USA), or 800 mcg of trivalent chromium with 12 mg of PE and 12 mg of SJ (Cr-800) once a day after breakfast for 12-weeks. Fasting blood, DEXA body composition, platelet aggregation, and ultrasound flow-mediated dilation studies were obtained at 0, 6, and 12 weeks of supplementation. Data were analyzed using general linear model (GLM) multivariate and univariate analysis with repeated measures, pairwise comparisons, and mean changes from baseline with 95% confidence intervals (CI). Differences with *p* < 0.05 were considered statistically significant, while differences with *p* > 0.05 to *p < *0.10 with medium (0.06) to large (>0.14) partial eta squared (η_p_^2^) effect sizes considered statistical tendencies. Means and 95% CI’s completely above or below baseline were considered clinically significant.

**Results**: Compared to the PLA group, there was evidence that Cr-400 ingestion promoted a significant reduction in VLDL cholesterol, triglycerides, insulin, and HOMA_IR_. (See Figure 1). Cr-800 supplementation promoted more optimal changes in body composition (greater gains in fat free mass and fat loss). There was some evidence that Cr-400 and Cr-800 supplementation significantly reduced VLDL-cholesterol and triglycerides after 4-weeks while HDL-cholesterol increased after 12 weeks with Cr-800 supplementation. Platelet aggregation increased above baseline and was significantly greater than PL with Cr-800 at 6 weeks (0.86 ohms [0.018, 1.71], *p* = 0.046) but not 12 weeks. After 12 weeks, pulse wave velocity was not significantly changed from baseline in any group although values tended to lower with Cr-800 compared to Cr-400. There was evidence that the Cr-400 dose had a greater impact on improving insulin sensitivity than Cr-800, but Cr-800 ingestion promoted greater fat loss and increases in fat free mass compared to Cr-400 and the PLA.

**Conclusions**: Results indicate that overweight and sedentary men and women initiating an exercise and weight loss program who supplement their diet with Cr, PE, and SJ observe greater improvement in insulin sensitivity (Cr-400), body composition (Cr-800), and blood lipid profiles (Cr-400 and Cr-800).

**Figure 1.**
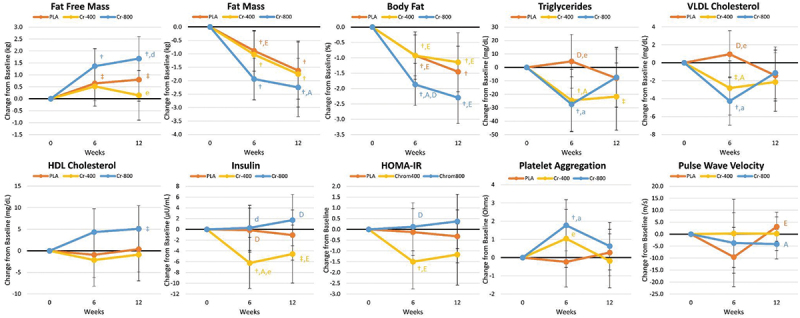
Changes in selected variables for the PLA, Cr-400, and Cr-800 groups. Data are means and ± 95% confidence intervals. † = *p* < 0.05 (‡*p* > 0.05 to *p* < 0.10) difference from baseline value. a = *p* < 0.05 (A = *p* > 0.05 to *p* < 0.10) difference from PLA, d = *p* < 0.05 (D = *p* > 0.05 to *p* < 0.10) difference from Cr-400, e = *p* < 0.05 (E = *p* > 0.05 to *p* < 0.10) difference from Cr-800.

**Effects of *Phyllanthus emblica* extract supplementation on markers of cardiovascular health, fitness, and weight loss in men and women initiating an exercise and diet intervention program**

Jacob Broeckel, Victoria Martinez, Kay McAngus, Broderick Dickerson, Megan Leonard, Elena Chavez, Jisun Chun, Megan Lewis, Dante Xing, Drew Gonzalez, Choongsung Yoo, Joungbo Ko, Ryan Sowinski, Christopher J. Rasmussen, Richard B. Kreider, FISSN.

Exercise & Sport Nutrition Lab, Texas A&M University, College Station, TX 77843, USA

Corresponding Authors: rbkreider@tamu.edu

**Background**: An aqueous extract of *Phyllanthus emblica* fruit extract (PE) has been shown to reduce endothelial dysfunction, platelet aggregation, and elevated blood glucose and lipid levels in support of cardio-metabolic health. This study examined the effects of PE supplementation on weight loss, body composition, and cardiovascular risk markers in sedentary and overweight men and women initiating a supervised exercise and weight loss program.

**Methods**: 68 men and women (48.3 ± 10 yrs., 34.7 ± 6 kg/m^2^, 40.5 ± 8 % fat) participated in a randomized, placebo-controlled, parallel, and repeated measures intervention trial. Volunteers participated in a standardized resistance training (3 sessions/week) and aerobic training (3 x 30 min sessions/week) program while reducing energy intake by 5 kcals/kg/d. In a double-blind and randomized manner, participants were matched by age, sex, BMI, and body mass to supplement their diet with placebo (PLA), 500 mg of PE (PE-500, Capros™, New Brunswick, NJ, USA), or 1,000 mg/d of PE (PE-1000) once a day after breakfast for 12-weeks. Fasting blood, DEXA body composition, platelet aggregation, and ultrasound flow-mediated dilation (FMD) studies were obtained at 0, 6, and 12 weeks of supplementation. Data were analyzed using general linear model (GLM) multivariate and univariate analysis with repeated measures, pairwise comparisons, and mean changes from baseline with 95% confidence intervals (CI). Differences with *p* < 0.05 were considered statistically significant, while differences with *p* > 0.05 to *p < *0.10 with medium (0.06) to large (>0.14) partial eta squared (η_p_^2^) effect sizes considered statistical tendencies. Means and 95% CI’s completely above or below baseline were considered clinically significant (Figure 1).

**Results**: Exercise and reducing energy intake promoted positive changes in weight and body composition. Although no significant differences were observed between groups, weight loss was more rapid with PE-500, fat free mass only increased above baseline with PE-1000 and changes in percent body fat were more favorable with PE-1000 supplementation. PE-500 promoted a significant reduction in total cholesterol after 6 weeks, while changes in VLDL-cholesterol and triglycerides were more favorable with PE-500 and PE-1000 ingestion. PE-1000 ingestion promoted more favorable changes in glucose, insulin, the glucose-to-insulin ratio, and HOMA-IR. Platelet aggregation (ohms) increased from baseline with PE-1000, while no effects were seen in FMD analysis.

**Conclusions**: Results indicate that overweight and sedentary men and women initiating an exercise and weight loss program who supplement their diet with PE observed some evidence (p < 0.05 or p > 0.05 to p = 0.10 trends from baseline or between groups) of more favorable changes in body composition, blood lipids, and insulin sensitivity. Additional research is needed to examine the potential health and performance effects of PE supplementation during training.

**Figure 1.**
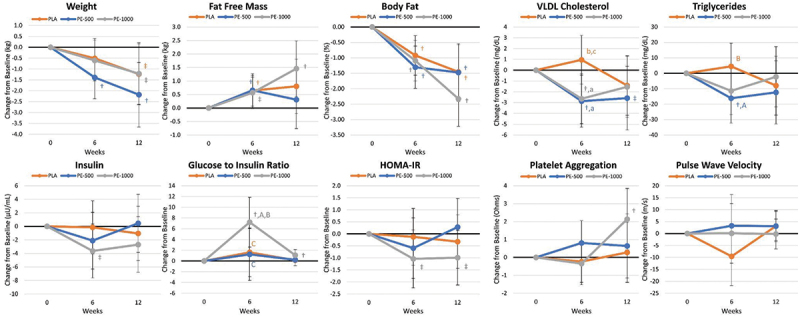
Changes in selected variables for the PLA, PE-500, and PE-1000 groups. Data are means and ± 95% confidence intervals. † = *p* < 0.05 (‡*p* > 0.05 to *p* < 0.10) difference from baseline value. a = *p* < 0.05 (A = *p* > 0.05 to *p* < 0.10) difference from PLA, b = *p* < 0.05 (B = *p* > 0.05 to *p* < 0.10) difference from PE-500, c = *p* < 0.05 (CE = *p* > 0.05 to *p* < 0.10) difference from PE-1000.

**Acknowledgments**

This study was funded by a grant from Natreon, Inc. (New Brunswick, NJ, USA) and Kerry (Naas, IRE) who acquired Natron in 2022 and funded the completion of the study.

**Placebo Supplementation for Promoting Weight Loss in Overweight and Obese Young Women**

Paulo R. V. Gentil^c^, Gislene B. Lima^a^, Victor S. Coswig^b^, Maria S. Silva^c^, Gustavo D. Pimentel^a^; Camila S. Seguro^a^, Carini S. and Silva^c^, Antonio Paoli^d^

^a^Federal University of Goiás – Faculty of Nutrition – Goiânia, Goiás, Brazil; ^b^Institute of Physical Education and Sports – Federal University of Ceara – Fortaleza, Ceará, Brazil; ^c^Federal University of Goiás – Faculty of Phisycal Education and Dance, Goiânia – Goiás, Brazil; ^d^Department of Biomedical Sciences – University of Padova, Padova – Veneto – Italy.

Corresponding Authors: paulogentil@gmail.com

**Background**: The primary aim of this study was to evaluate the effects of a placebo on the body composition of overweight and obese women participating in a program involving physical activity and nutritional counseling.

**Methods**: This study was a randomized clinical trial with 28 women allocated to three groups according to the expectation of treatment with supplements:100%, 50%, and 0% expectation of taking supplements that would increase metabolism. All volunteers performed high-intensity interval training for eight weeks (three times a week) and received nutritional guidance to change their eating habits.

**Results**: The results show that there was an increase in VO2max at the end of the intervention in the 100% (p = 0.021) and 0% (p = 0.035) groups. Total energy consumption did not change in any group, and there were no differences in body composition within or between the groups.

**Conclusions**: Placebo supplementation did not influence the reduction in weight and fat mass in overweight young adult women.

**Acknowledgments**: The University of Goiás for research support and Goiás Research Support Foundation (FAPEG) for the postgraduate scholarship. The funder played no role in data acquisition, data management, nor data interpretation.

**Impact of LeanBiome® intake on glycemic response, satiation, and gut microbiome diversity in healthy adults**

Zac Sinderman^a^, Oana A^b^, Enzver Keleszade, Sofia Kolida^a^, Adele Costabile

^a^OptiBiotix Health Plc. Innovation Center, Innovation Way, Heslington, York, Y010 5DG, UK; ^b^Department of Life Sciences, University of Roehampton, United Kingdom

Corresponding Authors: Zac Sniderman, zsniderman@optibiotix.com

**Background**: The aim of this study was to investigate the impact of the intake of LeanBiome®, (chromium enriched glucomannan-fructooligosaccharide complex) on glycemic and insulin response, satiation, and hunger biomarkers in healthy adults. LeanBiome® has been shown to support body fat reduction and to promote gut microbiome diversity, while reducing fat intake, and food cravings in human intervention studies.

**Methods**: Using a double-blind, placebo-controlled, crossover study design we assessed the acute impact of the intake of a single LeanBiome® dose in 20 healthy adults (10 males, 10 females; BMI 18.5-24.9 kg/m^2^, 18-65 years; fasting blood glucose 4-7 mM), undergoing an oral glucose tolerance test. Volunteers ingested 50 g of available carbohydrates (dextrose) and 50 g dextrose supplemented with 3 g LeanBiome® (LBdextrose) on separate days. Test interventions were blind packaged and dissolved in 250 ml water. Blood samples were obtained at 0 min (fasting) 15, 30, 45, 60, 75, 90, 120 min and 150 min post intervention intake. Feelings of hunger, fullness, desire to eat, and amount of food desired were assessed at 0, 75 and 150 min using the visual analogue scale. The impact of daily LeanBiome® intake over a 4-week period as part of a calorie-controlled diet program was then assessed in 12 overweight and obese adults (18-65 years, BMI 25-35 kg/m^2^). Anthropometric parameters, mood and cravings were recorded, and fecal samples were collected for metagenomic analysis. All studies were conducted according to SPIRIT requirements and CONSORT guidelines and have been approved by the University of Roehampton Research Ethics Committee (LSC 18/238).

**Results**: LBdextrose intake reduced both the glucose and insulin peak when compared to the intake of dextrose alone. Volunteer scores on appetite related parameters did not differ significantly between dextrose and the LBdextrose tests at baseline. At 75 min the feeling of fullness was significantly higher in the SBdextrose compared to dextrose alone (P < 0.05), while the desire to eat was significantly lower compared to dextrose. At 150 min, although hunger scores increased compared to baseline for both tests, SBdextrose scores remained significantly lower compared to dextrose (P < 0.05). Similar effects were noted for desire to eat, and the amount of food volunteers felt they could eat. Fullness for the SBdextrose was significantly higher (P < 0.05) compared to dextrose and the baseline values.

LeanBiome® intake over a 4-week period resulted in statistically significant reductions (P < 0.01) in weight, waist and hip circumference, percentage body fat, fat mass, cravings for savory and sweet foods, combined with significant improvements in mood. Statistically significant increases were also observed in gut microbiome diversity and the relative abundance *Bifidobacterium* and groups associated with lean body weight Christensenellaceae and Bacteroidetes.

**Conclusions**: The findings of the study suggest that LeanBiome® can potentiate insulin response and have an acute effect on feelings of hunger and fullness over a prolonged period, even when combined with glycemic food ingredients. Long term intake can improve body composition through supporting healthy eating habits, managing cravings for sweet and savory foods, and reducing hunger, while improving mood and gut microbiome composition and diversity.

**Acknowledgments**: This research was funded by OptiBiotix Health Ltd. The funder played no role in data acquisition, data management, nor data interpretation.

**Impact of Powdered Tart Cherry Supplementation on Performance and Recovery Following Repeated Sprint Exercise**

Alex C. Schrautemeier^a^, Anthony M. Hagele^a^, Kevin F. Holley^a^, Joesi M. Krieger^a^, Joshua M. Ionnatti^a^, Connor J. Gaige^a^, Ralf Jäger^b^, Chad M. Kerksick, FISSN^a^

^a^Exercise and Performance Nutrition Laboratory, Department of Kinesiology, Lindenwood University, St. Charles, MO, USA; ^2^Increnovo, Whitefish Bay, WI USA

*Corresponding Authors: ckerksick@lindenwood.edu

**Background**: Due to its high polyphenol content and the purported ability to function in an anti-inflammatory and anti-oxidative manner, tart cherry supplementation has been proposed to enhance athletic performance. This study examined the effects of powdered tart cherries on various performance metrics following a repeated sprint exercise protocol in physically active men and women.

**Methods**: 40 (18 M, 22 F) healthy, active participants (24.6 ± 5.5 yrs, 171.5 ± 11 cm, 71.7 ± 14.5 kg, 24.2 ± 3.1 kg/m^2^) participated in this randomized, double-blind, placebo-controlled, parallel study design. Placebo (PLA) or tart cherry (TC) supplementation (500 mg/day) occurred for a total of ten days comprising of the seven days prior to, day of, and two days following a repeated sprint exercise bout (15 x 30-meter sprints with a 10-meter deceleration with 1 minute rest between sprints). Performance was assessed through countermovement jumps and isokinetic knee extension at 180°/s. Recovery was evaluated using visual analog scales for soreness, recovery, and readiness to train. Muscle damage was evaluated using creatine kinase. These measures were evaluated at baseline, and at 1 hour, 24 hours, and 48 hours post-exercise.

**Results**: Significant main effects of time were observed with recovery VAS (*p* < 0.001), readiness to train VAS (*p* < 0.001), and jump height (p = 0.014) experiencing similar reductions while soreness VAS (*p* < 0.001) and creatine kinase (*p* = 0.05) experiencing similar increases in response to the repeated sprint protocol and supplementation. No significant group x time differences were observed for jump height (PLA:-6.7 ± 10.4% vs. TC: −11.0 ± 17.9%, *p* = 0.608), peak propulsive force (PLA: 0.25 ± 4.62% vs. TC: 2.20 ± 7.39%, *p* = 0.194), knee extension peak torque at 180°/s (PLA: 10.49 ± 73.53% vs. TC: −1.04 ± 49.56%, *p* = 0.335), readiness to train VAS (PLA: −23.0 ± 19.2% vs. TC: −14.7 ± 20.2%, *p* = 0.401), soreness VAS (PLA: 249.6 ± 323.3% vs. TC: 260.9 ± 432.1%, *p* = 0.838), recovery VAS (PLA: −24.6 ± 17.9% vs. TC: −8.2 ± 40.5%, *p* = 0.251), and creatine kinase (PLA: 22.8 ± 35.5% vs. TC: 90.4 ± 225.6%, *p* = 0.31).

**Conclusions**: Following a repeated sprint protocol, significant changes in perceived recovery, readiness to train, soreness, jump height, and creatine kinase were observed in both experimental groups. No statistically significant differences were observed between PLA and TC for their ability to impact recovery and performance outcomes measured in this study.

**Acknowledgements**: This study was funded by an unrestricted grant from Increnovo (Whitefish Bay, WI USA).

**Conflicts of Interest**: The authors have no conflicts of interest to declare.

**Energy Expenditure and Fat Oxidation Changes Following 28-Days of Consumption of a Caffeinated Energy Drink**

Anthony M. Hagele^a^, Joesi M. Krieger^a^, Connor J. Gaige^a^, Alex C. Schrautemeier^a^, Joshua M. Iannotti^a^, Olivia J. Mennemeyer^a^, Chad M. Kerksick^a^, FISSN, Petey W. Mumford^a^

^a^Exercise and Performance Nutrition Laboratory, Department of Kinesiology, Lindenwood University, St. Charles, MO, USA

*CONTACT: ckerksick@lindenwood.edu

**Purpose**: The purpose of this study was to evaluate the effects of 28 days of supplementation with a caffeine-based energy drink on energy expenditure and fat oxidation in healthy adults.

**Methods**: In a randomized, double-blind, placebo-controlled trial, 60 participants (33 males, 27 females; 27 ± 8 years, 173.6 ± 10.4 cm, 80.9 ± 12.35 kg; 26.7 ± 2.18 kg/m^2^) consumed a daily dose of either a caffeine-based (200 mg) energy drink (CAF) (ASHOC Energy, Newport Beach, CA, USA) or placebo (PLA) for 28 days. Indirect calorimetry was performed 0, 30, 60, 90, and 120 minutes (min) after ingestion on Day 1 and Day 28 of ingestion to determine rates of energy expenditure and fat oxidation.

**Results**: After 28 days of consumption, CAF significantly increased energy expenditure from baseline (1985 ± 293.24) compared to 30 (2101 ± 290.46), 60 (2025 ± 257.12), and 90 (2046 ± 283.54) minutes (all p < 0.05). When comparing the groups, CAF consumption increased energy expenditure in response to baseline values to a greater magnitude at 30, 60, and 90 minutes (all p < 0.05). When evaluating the long-term dosing effects between the groups, CAF had higher energy expenditure change scores (Day 28 – Day 0) at baseline (CAF: 72.04 ± 172.34, PLA: −11.51 ± 185.54; p = 0.031), and trended higher at 30 (CAF: 76.81 ± 181.15, PLA: −8.24 ± 157.63; p = 0.057) and 90 minutes (CAF: 76.67 ± 190.95, PLA: 5.50 ± 176.37; p = 0.051). After 28 days of consumption, both CAF and PLA groups showed increased fat oxidation at 60 (CAF: 190.55 ± 56.69, PLA: 194.90 ± 48.78), 90 (CAF: 182.27 ± 48.62, PLA: 183.65 ± 40.89), and 120 (CAF: 188.12 ± 52.98, PLA: 190.54 ± 42.29) minutes (all p < 0.001) compared to their respective baseline values (CAF: 159.59 ± 63.13, PLA: 185.72 ± 59.99). When comparing the groups, a trend (p = 0.052) for higher fat oxidation at 120 minutes was exhibited in the CAF group (CAF: 11.55 ± 56.45, PLA: 0.82 ± 57.32). No differences (p > 0.05) in fat oxidation change scores were observed between groups.

**Conclusions**: This study demonstrated that energy expenditure and fat oxidation rates were significantly increased in response to ingestion after 28 consecutive days of supplementation. Moreover, this data also illustrates that a successful sustainment of increased energy expenditure rates was achieved with caffeine ingestion after 28 days of consumption in healthy adults. Collectively, these findings suggest that regular consumption of CAF for 28 days enhances metabolic rate and fat oxidation. The extent to which these changes may have additional impact on weight management and metabolic health requires further investigation. Further research is needed to explore the long-term effects and potential mechanisms underlying these changes.

**Acknowledgements**: This study was funded by an unrestricted grant A SHOC Beverage, LLC (Newport Beach, CA)

**Conflicts of Interest**: The authors have no conflicts of interest to declare.

**Acute Supplementation with a Caffeinated Energy Drink on Energy Expenditure And Fat Oxidation**

Joesi M. Krieger^a,^ Alex C. Schrautemeier^a,^ Anthony M. Hagele^a^, Connor J. Gaige^a^, Olivia J. Mennemeyer^a^, Sydney K. Tolbert^a^, Joshua M. Iannotti^a^, Chad M. Kerksick, FISSN^a^, Petey W. Mumford^a^

^a^Exercise and Performance Nutrition Laboratory, Department of Kinesiology, Lindenwood University, St. Charles, MO, USA

*Corresponding Authors: ckerksick@lindenwood.edu

**Purpose**: The purpose of this study was to evaluate the effects of acute supplementation of a caffeine-based energy drink on energy expenditure and fat oxidation.

**Methods**: In a randomized, double-blind, placebo-controlled study, 33 males and 27 females (n = 60, 27 ± 8 years, 173.6 ± 10.4 cm, 80.9 ± 12.4 kg, 26.7 ± 2.2 m/kg2) consumed a single dose of either a caffeine-based (200 mg) energy drink (CAF) (ASHOC Energy, Newport Beach, CA, USA) or placebo (PLA). Indirect calorimetry was performed 0, 30, 60, 90, and 120 minutes (min) after ingestion to quantify resting energy expenditure and fat oxidation.

**Results**: CAF ingestion significantly increased baseline (1930 ± 354.12) energy expenditure at 30 (2025 ± 353.49), 90 (1970 ± 343.09), and 120 (1983 ± 373.05) minutes (all p < 0.05). However, following PLA ingestion energy expenditure significantly decreased from baseline (1936 ± 308.56) at 60 (1864 ± 261.95), 90 (1894 ± 285.88), and 120 (1878 ± 289.55) minutes (all p < 0.05). When comparing the groups, CAF consumption increased energy expenditure in response to baseline values to a greater magnitude at all time points (all p < 0.01). Rates of fat oxidation increased after CAF ingestion from baseline (159.59 ± 63.13) compared to 60 (190.55 ± 56.69), 90 (182.27 ± 48.62), and 120 (188.12 ± 52.98) minutes (p < 0.005), while PLA baseline (185.72 ± 59.99) significantly decreased 30 (168.41 ± 48.75) minutes after ingestion (p = 0.001). Compared to PLA, CAF ingestion was associated with greater increases in fat oxidation 60, 90, and 120 minutes of ingestion (p < 0.02).

**Conclusion**: This study demonstrated that a single dose of a caffeine-based energy drink containing 200 mg of caffeine significantly increased energy expenditure and fat oxidation in healthy males and females. These findings suggest that a single dose of a CAF energy drink can acutely instigate changes in metabolic rate and fat oxidation. Further research is needed to explore the impacts with sustained use as well as the long-term effects and underlying mechanisms.

**Acknowledgements**: This study was funded by an unrestricted grant A SHOC Beverage, LLC (Newport Beach, CA)

**Conflicts of Interest**: The authors have no conflicts of interest to declare.
